# Photocatalytic degradation of dissolved organic matter under ZnO-catalyzed artificial sunlight irradiation system

**DOI:** 10.1038/s41598-020-69115-7

**Published:** 2020-08-04

**Authors:** Thao Thi Nguyen, Seong-Nam Nam, Jungryul Kim, Jeill Oh

**Affiliations:** Department of Civil and Environmental Engineering, Chung-Ang University, 84 Heukseok-ro, Dongjak-gu, Seoul, 06974 Republic of Korea

**Keywords:** Biogeochemistry, Environmental sciences

## Abstract

This study investigates the photocatalytic degradation of dissolved organic matter (DOM) under ZnO-assisted artificial sunlight system at various conditions (ZnO dosage, pH, and the presence of Cl^−^, SO_4_^2−^, and HCO_3_^−^). The results show that the degradation of DOM follows a pseudo-first-order kinetics. Fluorescence excitation–emission matrices coupled with parallel factor (EEM-PARAFAC) analysis decomposes DOM into two fluorophores (C1 and C2). The total removals and photodegradation rates calculated with DOC, UV_254_, and the F_max_ of C1 are similar, increasing with higher ZnO dosages and being highest in pH 7 and lowest in pH 4. ZnO dosage has a similar effect on DOM degradation when assessed using C2, as with C1, but pH effect is not consistent. As for the anions, HCO_3_^−^ shows the strongest inhibition for DOC, UV_254_ and C1 while Cl^−^ has the strongest facilitation effect for C2. The total removal and photodegradation rates calculated with the F_max_ of C1 and C2 are higher than those calculated using DOC and UV_254_. This study demonstrates that the successful application of EEM-PARAFAC analysis in addition to traditional parameters can provide further insight into the photocatalytic degradation mechanisms associated with DOM in conjunction with a ZnO catalyst under artificial sunlight.

## Introduction

Dissolved organic matter (DOM) is a heterogeneous mixture of aliphatic and aromatic polymers containing oxygen, nitrogen, and sulfur functional groups. DOM plays an important role in both natural and engineered water systems. The presence of DOM in aquatic environments can cause various problems such as the adsorption and deposition of organic foulants in membrane treatment processes and the formation of disinfection by-products (DBPs)^1–3^. Humic acids (HAs), which are the main contributors to DOM, are well-known as a precursor to carcinogenic and mutagenic DBPs, such as trihalomethanes and haloacetic acid^4–6^. HAs also turn water a brownish‐yellow color, form complex species with metals and pesticides, increase the chlorine demand of water, cause corrosion in pipelines, and foul and plug membranes^4–6^. The effective removal of DOM from water and wastewater is thus an important treatment objective and a major issue for water and wastewater treatment plants worldwide.

Different treatment methods can be applied depending on the characteristics of the DOM, with the most common methods for DOM removal being coagulation, adsorption, membrane filtration, biological, ion exchange processes, and advanced oxidation processes (AOPs)^1–3^. Of these methods, AOPs have been found to be particularly efficient. AOPs rely on the in-situ production of highly reactive hydroxyl radicals (^·^OH) with the help of one or more primary oxidants (e.g., ozone, hydrogen peroxide, or oxygen), energy sources (e.g. ultraviolet, solar, or visible light), and/or catalysts (e.g. WO_3_, ZnO, or TiO_2_)^2^. Refractory pollutants in water react with ^·^OH, leading to its decomposition or mineralization into CO_2_, H_2_O, and inorganic ions^2^. Of the various combinations of oxidants that have been developed for DOM removal, heterogeneous photocatalysis has shown considerable potential as a versatile, low-cost, environmentally friendly, and sustainable AOP treatment technology.

Heterogeneous photocatalysis involves the irradiation of semiconductor catalysts (e.g., TiO_2_, ZnO, WO_3_, Fe_2_O_3_, CdO, CdS, SnO_2_, etc.) with a light source (e.g., ultraviolet (UV), sunlight, or artificial light) to generate highly reactive oxygen species (ROS) (e.g., ^·^OH,${O}_{2}^{\cdot-}$) for the subsequent mineralization of organic pollutants. When a semiconductor catalyst is photoinduced, electrons in the valence band (VB) are transferred to the conduction band (CB), generating an electron–hole pair (${e}_{CB}^{\_}/{h}_{VB}^{+}$). This leads to the formation of ^·^OH via oxidation from the reactions between H_2_O or OH^−^ molecules and VB holes (${h}_{VB}^{+}$), while superoxide radicals ${(O}_{2}^{\cdot-})$ from the dissolved O_2_ and CB electrons (${e}_{CB}^{\_}$) are formed via reduction^2–9^. These ROS are then able to completely break down organic contaminants into CO_2_, H_2_O and other inorganic substances^7–9^. According to recent researches, the heterogeneous photocatalysis is one of the most suitable treatment technologies to degrade DOM and reduce the formation of DBPs^2–4^. There are three pathways for the removal of DOM, namely, oxidation by ^·^OH, reduction by ${O}_{2}^{\cdot-}$, and adsorption by catalyst^2–4^. The majority of previously proposed DOM photodegradation approaches have harnessed the direct excitation of molecules using UV light (e.g., UV/TiO_2_ and UV/ZnO)^3, 10^. However, UV radiation only accounts for 5–7% of sunlight, which substantially limits the practical application of the UV photocatalytic oxidation technology. There is a remaining research question on how to exploit solar radiation (sunlight), a renewable, abundant, non-polluting, and cheap energy source^9^, with a highly efficient and stable photocatalyst to enhance the photocatalytic efficiency and promote the application of photocatalytic technology for the treatment of water and wastewater.

Along with TiO_2_ and modified-TiO_2_^3^, visible/solar photocatalytic studies using ZnO has been reported to lead to the high-performance of photocatalysis of organic compounds in aqueous solutions. ZnO is a wide-band-gap semiconductor with a large excitation binding energy of 60 meV at room temperature. It exhibits great potential for use in photodegradation due to its low-cost, non-toxicity, strong oxidation ability, and suitable photocatalytic properties^11^. ZnO is also more efficient in terms of its absorption of a broad range of the solar spectrum compared to TiO_2_^11^.

Fluorescence spectroscopy is a non-destructive, reliable, and highly sensitive optical technique for rapidly assessing DOM levels in aquatic environments^12–14^. Fluorescence excitation–emission matrices (EEMs) combined with parallel factor (PARAFAC) analysis have proven particularly useful over the last decade in tracking various DOM substances in water environments^14^. The location of the peaks and intensities of individual PARAFAC components can be used to evaluate water quality and treatment performance^13^. Recently, EEM-PARAFAC analysis has been applied to monitor changes in DOM fluorophores in UVA/TiO_2_ systems, thus providing new insight into the photocatalytic degradation of DOM^15–17^. Therefore, in addition to traditional parameters (e.g., UV absorbance at 254 nm, DOC, SUVA, etc.), EEM-PARAFAC analysis was employed in the present study to monitor the photodegradation of DOM within a ZnO-catalyzed artificial sunlight system.

The objectives of this study were as follows:

1. To compare photocatalytic changes in DOM under different experiment conditions (i.e., with changes in ZnO dosage, pH, and presence of inorganic anions).
2. To track the behavior of individual DOM fluorophores identified using the EEM-PARAFAC approach during the photocatalytic degradation process.
3. To assess the photocatalytic degradation mechanisms and compare the photolysis, photocatalysis, and adsorption of DOM.

## Experimental

### Chemicals and reagents

Zinc oxide (CAS No. 1314-13-2, MF: ZnO, MW: 81.39 g/mol), sodium chloride (NaCl), calcium chloride (CaCl_2_), sodium bicarbonate (NaHCO_3_), and sodium sulfate (Na_2_SO_4_) were purchased from Sigma-Aldrich (USA). Sodium hydroxide (NaOH) was obtained from Daejung Chemicals (South Korea). Sulfuric acid (H_2_SO_4_, purity ≥ 96%) was purchased from Kanto Chemicals (Japan). Humic acid (HA) was obtained from Sigma-Aldrich (USA). The HA was dissolved in de-ionized water (DI; ≥ 18.2 Ω cm^−1^) and filtered through a 0.45-μm hydrophilic polytetrafluoroethylene (PTFE) membrane to produce a stock DOM solution with a concentration of DOC ≈ 10 mg/L. Other solutions were prepared using de-ionized water and diluted as required.

### Photocatalytic experiments

The solar irradiation setup consisted of a light source and a 100-mL glass reactor (Fig. S1)^9^. The light source was a 300-W Xenon lamp installed in a solar simulator (SLB300B, Sciencetech, Canada). All experiments were performed at room temperature (23 ± 1 °C for a 180-min reaction time). The solution in the reactor was gently stirred with a magnetic bar for uniform mixing. The initial pH levels of the solution (pH 4, 7, and 10) were adjusted by adding 1 M H_2_SO_4_ and 1 M NaOH, and ZnO was tested at dosages of 0.1, 0.2, and 0.3 g/L. For DOM analysis, samples were taken at regular time intervals (0, 30, 60, 120, and 180 min), and immediately filtered through 0.45-μm PTFE syringe filters to separate the ZnO powder from the solution.

Ionic species such as chloride, bicarbonate, and sulfate that are frequently present in surface water and wastewater may affect the efficiency of DOM degradation due to competitive reactions of those ions with ^·^OH^18^. Thus, in this study, the effect of anionic species, including Cl^−^, SO_4_^2–^, and HCO_3_^−^, on DOM photocatalysis was investigated, at concentrations of 10 mM (low) and 50 mM (high) by adding the required quantities of NaCl, Na_2_SO_4_, or NaHCO_3_ to the DOM solution.

### Analytical methods

DOM was characterized and monitored over the course of the photocatalytic degradation process using ultraviolet–visible (UV/Vis) absorbance and fluorescence spectroscopy. DOM levels during photocatalytic degradation were measured in terms of DOC, UV_254_, and SUVA_254_. DOC was determined using a TOC-V_CPH_ analyzer (Shimadzu, Japan). A standard solution for DOC calibration was prepared using potassium hydrogen phthalate (KHP) in the range of 1–20 mgC/L. UV/Vis spectra were recorded at a wavelength range of 200 nm to 800 nm using UV/Vis spectrophotometer (SPECORD 200 PLUS, Analytik Jena AG, Germany) with a 1-cm quartz cuvette. UV_254_, one of the most common parameters used to determine the degradation rate of DOM, is known to be related to DOM properties such as aromaticity and molecular weight^12^. Specific ultraviolet absorbance (SUVA_254_), which is calculated as UV_254_ × 100/DOC, describes the hydrophobicity and hydrophilicity of DOM in water. SUVA_254_ values greater than 4 indicate that the DOM contains mainly hydrophobic and especially aromatic compounds, while the values less than 3 indicate that the DOM consists mainly of hydrophilic compounds^12^. Fluorescence EEMs were constructed by scanning water samples over an excitation range of 230–450 nm in 1-nm increments and over an emission range of 260–550 nm in 1-nm increments using a Cary Eclipse fluorescence spectrophotometer (Agilent Technologies Inc., USA). The excitation and emission bandwidths were both set at 10 nm, and the scanning speed was set at 9,600 nm/min. To limit second-order Rayleigh scattering, a 290-nm cutoff was used for all samples. In order to minimize the inner filter effect, the samples were diluted close to ~ 1 mgC/L of DOC before EEM measurements were taken. The sample EEMs were subtracted by a water blank that was measured on the same day as the samples and were normalized by dividing the EEMs with the Raman peak area of 370–700 nm measured on the same day as the sample measurement.

### PARAFAC modeling

In PARAFAC analysis, the EEM dataset is decomposed into a set of trilinear terms (F) and a residual array, and it estimates the underlying EEM spectra by minimizing the sum of the squared residuals of the trilinear model as follows^14^:

1 $$x_{ijk} = \mathop \sum \limits_{f = 1}^{F} a_{if} b_{jf} c_{kf} + \varepsilon_{ijk} \;\;\;\;\;\;i = 1, \ldots , I;j = 1, \ldots , J;k = 1, \ldots , K,$$

where *x*_*ijk*_ is the fluorescence intensity for the *i*_*th*_ sample at emission wavelength *j* and excitation wavelength *k*, *a*_*if*_ is directly proportional to the concentration of the *f*_*th*_ fluorophore in the *i*_*th*_ sample (defined as scores), and *b*_*jf*_ and *c*_*kf*_ are the estimates of the emission and excitation spectra, respectively, for the *f*_*th*_ fluorophore. *F* represents the number of components in the model, and *ε*_*ijk*_ is the residual variability not accounted for by the model. PARAFAC modeling using MATLAB 7.0 (Mathworks, MA, USA) with the DOMFluor Toolbox (https://www.models.life.ku.dk) was conducted with two to seven components. The maximum fluorescence intensity (F_max_) of the individual components was used to represent their relative concentrations. The EEM data for the PARAFAC model were obtained based on 190 EEM data. The determination and validation of component in the model validated were performed using split-half validation, explained variation (> 99.9%), the core consistency diagnostic (> 85%), Tucker’s congruence coefficient, and the spectral analysis of the excitation and emission loadings^17^. The more details on the model are described in other study^19^.

## Results and discussion

### DOC changes under photocatalytic degradation

**Figure 1:**
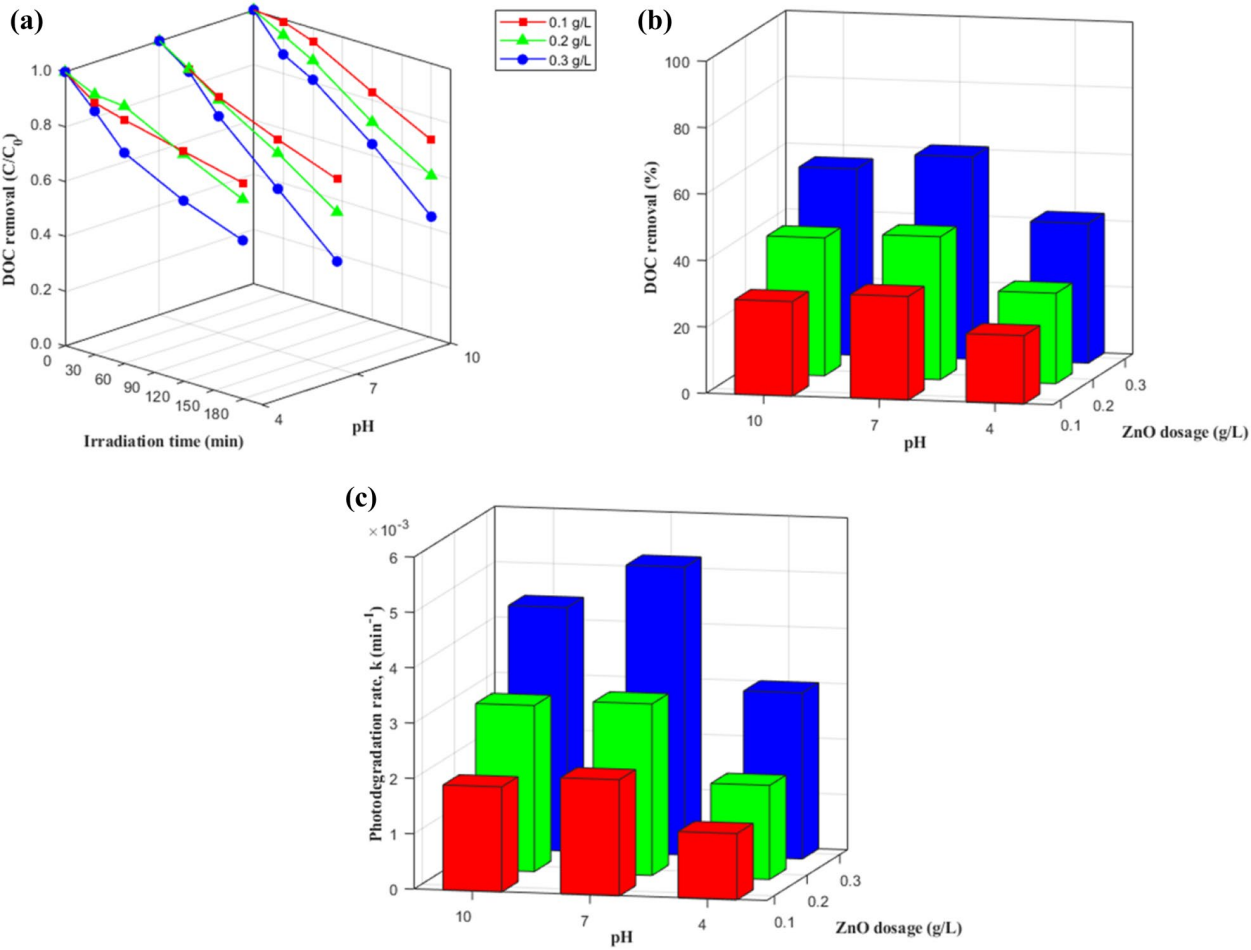
Effect of ZnO dosage and pH on DOC removal for photocatalytic degradation of DOM: (**a**) degradation curves, (**b**) removal %, and (**c**) degradation rates.

**Figure 2:**
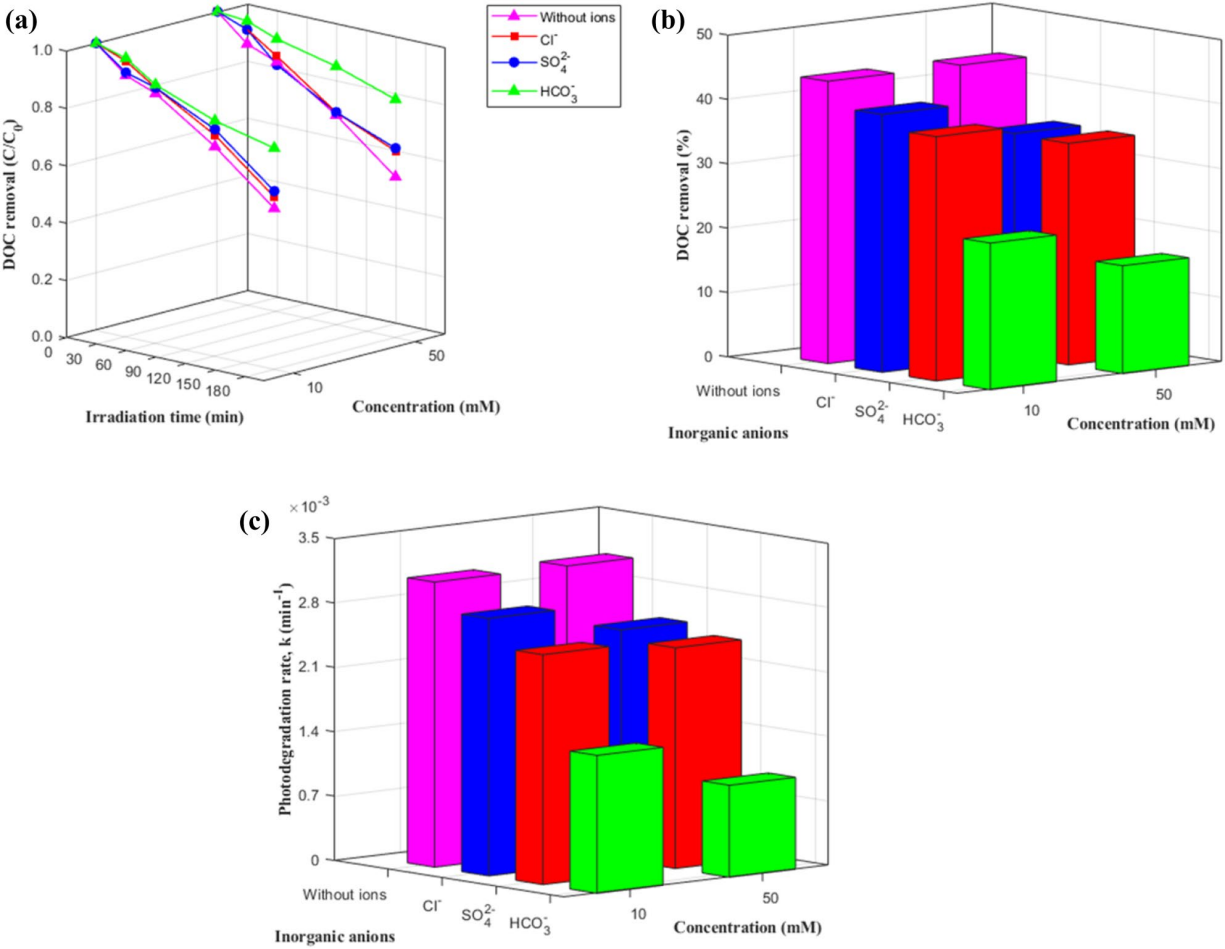
Effect of inorganic anions on DOC removal for photocatalytic degradation of DOM with 0.2 g/L ZnO at pH 7: (**a**) degradation curves, (**b**) removal %, and (**c**) degradation rates.

The changes in DOC during photocatalysis are illustrated in Figs. 1–2 and Table S1. DOC removal after 180-min irradiation varied from 16.76 to 60.88% depending on the experimental conditions. All observed degradation trends followed a pseudo-first-order kinetic model (R^2^ = 0.96–1.00), which has also been reported in other studies to describe the photodegradation of DOM^15–17^. The effects of ZnO dosage, pH level, and presence of inorganic anions on the degradation of DOM are described in detail in the following sub-sections.

#### Effect of ZnO dosage

The relationship between ZnO dosage and DOC degradation is presented in Fig. 1 and Table S1. When ZnO dosage increased from 0.1 to 0.3 g/L, DOC removal and apparent degradation rate (*k*_*app*_) were ∆ 21.42% and 2.49-fold higher at pH 4, ∆ 29.64% and 2.53-fold higher at pH 7, and ∆ 27.62% and 2.28-fold higher at pH 10, respectively. It is assumed that more active sites become available with increasing ZnO dosage, thus facilitating the generation of OH and consequently greater DOC removal and a higher degradation rate.

#### Effect of pH

As illustrated in Fig. 1 and Table S1, DOC removal and *k*_*app*_ were highest at a pH of 7 for all ZnO dosage levels. This observation could be explained by the ionization of DOM and the zeta potential (ZP) of ZnO at different pH levels. The acidic functional groups (e.g., –COOH and –OH_phenolic_) in HA molecules would become more ionized as the aqueous pH increases because pK_a,-COOH_ and pK_a,-OH_ have been reported to be 4.7 and 12.5, respectively (Eqs. 2)^20^:

2 $${\text{HOOC}} \cdots -{\text{HA}}- \cdots {\text{OH}} \to^{ - } {\text{OOC}} \cdots- {\text{HA}} - \cdots {\text{O}}^{ - } \, + \, 2{\text{H}}^{ + }$$

HAs are negatively charged over a wide pH range (2.0–10.7) ^21^, while the ZP of ZnO is positive at a pH range of 6.7–9.3 and negative otherwise (the pH_ZPC_ of ZnO = 9.0 ± 0.3) ^22^. Thus, at pH 7, where the dominant charges of ZnO and HA oppose each other, the electrostatic attraction between HA molecules and the ZnO surface would lead to the more rapid exposure of the HAs to reactive species (especially ^·^OH), resulting in the maximum DOC removal and photodegradation rate. On the other hand, at a pH of 4 and 10, both the HAs and ZnO are negatively charged, thus the repulsive force between the HA molecules and the ZnO surface would be strong. Therefore, there would be limited opportunities for the HA molecules to contact with reactive species near the ZnO surface, reducing removal and *k*_*app*_.

In addition, it was found in the present study that DOC removal was always higher at pH 10 than at pH 4 for the same ZnO dosage. It has been reported that ZnO aggregation occurs at pH 4 (pH_ZPC_ of ZnO = 9.0 ± 0.3)^22^, which would slow the mass transport rate and consequently reduce the active surface area of ZnO. In addition, acidic conditions (i.e., less OH^−^) are less favorable for the formation of ^·^OH via the hole oxidation of OH^−^, lowering the efficiency of the attack of ^·^OH on DOM and the photocatalytic oxidation rate^15, 16^. These two reasons lead to lower total DOC removal and a lower photodegradation rate at a pH of 4.

#### Effect of inorganic anions

Figure 2 and Table S1 show the effects of inorganic ions on DOC removal. When fitting a pseudo-first-order kinetics model (R^2^ = 0.96–0.99), the presence of Cl^−^, SO_4_^2−^, and HCO_3_^−^ anions inhibited DOC removal. This occurred possibly because of two reasons. First, the ZnO surface is positively charged at pH 7, and the anions can be easily adsorbed onto the positively charged surface of the catalyst by electrostatic attraction, leading to the competitive adsorption. Second, the anions acted as free radical scavengers by reducing the availability of positive holes and by competitively reacting with ^·^OH^6, 23–25^, as given by the following reactions (Eq. 3–10) (Table S2):

3 $${\text{Cl}}^{ - } + {\text{h}}_{{{\text{VB}}}}^{ + } \to {\text{ Cl}}^{ \cdot }$$

4 $${\text{Cl}}^{ - } + { }^{ \cdot } {\text{OH}} \to {\text{ HOCl}}^{ \cdot - } \;\;\;\;\;\;\;\;\;{ }\left( {k = 4.3 \times 10^{9} {\text{ M}}^{ - 1} {\text{s}}^{ - 1} } \right)$$

5 $${\text{HOCl}}^{ \cdot - } \to {\text{Cl}}^{ - } + { }^{ \cdot } {\text{OH }}\;\;\;\;\;\;\;\; \left( {k = 6.1 \times 10^{9} {\text{ M}}^{ - 1} {\text{s}}^{ - 1} } \right)$$

6 $${\text{SO}}_{4}^{2 - } + {\text{ h}}_{{{\text{VB}}}}^{ + } { } \to {\text{ SO}}_{4}^{ \cdot - }$$

7 $${\text{SO}}_{4}^{2 - } +^{ \cdot } {\text{OH}} \to {\text{ SO}}_{4}^{ \cdot - } + {\text{ OH}}^{ - } { }\left( {k = 1.18 \times 10^{6} {\text{ M}}^{ - 1} {\text{s}}^{ - 1} } \right)$$

8 $${\text{HCO}}_{3}^{ - } + {\text{h}}_{{{\text{VB}}}}^{ + } \to {\text{CO}}_{3}^{ \cdot - } + {\text{ H}}_{2} {\text{O}}$$

9 $${\text{HCO}}_{3}^{ - } +^{ \cdot } {\text{OH}} \to {\text{ CO}}_{3}^{ \cdot - } + {\text{ H}}_{2} {\text{O }}\left( {k = 8.5 \times 10^{6} {\text{ M}}^{ - 1} {\text{s}}^{ - 1} } \right)$$

The strength of the inhibition effect followed the order of HCO_3_^−^ > SO_4_^2−^ > Cl^−^ > no ions, possibly because HCO_3_^−^ had the strongest capturing effect on ^·^OH (*k* = 8.5 × 10^6^)^25^. The HCO_3_^−^ quenched the ${h}_{VB}^{+}$, which prevented the generation of ^·^OH (i.e., it inhibited the ${h}_{VB}^{+}$ + H_2_O → ^·^OH + H^+^ reaction) and may, in turn, have led to the formation of ${CO}_{3}^{\cdot-}$ via the oxidation of ${HCO}_{3}^{\cdot-}$ by ${h}_{VB}^{+}$ (Eq. 8), with a lower reactivity (E° = 1.78 V) than ^·^OH^26^. ${CO}_{3}^{\cdot-}$ has a weaker oxidative ability than ^·^OH and rarely reacts with organic matter, thus decreasing the reaction rate significantly^6^. In addition, HCO_3_^-^ anions form a strong combination on the surface of the catalyst and can significantly inhibit the adsorption of HAs on the catalyst due to the weak absorption competition between HCO_3_^−^ and HAs^6^.

### Change in UV_254_

The degradation process as measured using UV_254_ is summarized in Figs. S2, S3 and Table S1. All photodegradation rates fit a pseudo-first-order kinetics model (R^2^ = 0.93–1.00), and there was a strong correlation between UV_254_ and DOC (R^2^ = 0.92–0.98), suggesting that chromophoric DOM accounted for the most significant proportion of DOC removal^16^. The effects of ZnO dosage, pH level (Fig. S2), and the presence of inorganic anions (Fig. S3) on UV_254_ removal and photodegradation rate were analogous to those described in the previous section. The highest in UV_254_ removal was 96.54% after 180 min of irradiation with a ZnO dosage of 0.3 g/L, a pH of 7, and no additional inorganic anions.

Total removal and the photodegradation rate calculated based on UV_254_ were much higher than those calculated using DOC concentration under all experimental conditions, which may be because the terminal functional groups of the aromatic compounds (e.g., hydroxyl and carboxyl) reinforced the adoption affinity of the surface of the catalyst particles^15–17^ and/or some of the DOM chromophores were partially transformed into non-UV-absorbing compounds (e.g., low-molecular-weight organic acids, alcohols, etc.) in the photochemical reaction^13, 24^.

The rapid reduction in UV_254_ with irradiation time (Fig. S2) suggests that the DOM chromophores, which mostly consisted of large aromatic rings, might have been rapidly broken down into smaller non-aromatic structures^12, 25^. The UV/Vis absorption spectra of DOM showed, as expected, rapid decrease with reaction time, and the remained absorption in UV range, even after 180-min irradiation implies the necessity of experimental optimizations (such as reaction time, power of light source, dosage of catalyst, etc.) for complete mineralization.

### Change in SUVA_254_

**Figure 3:**
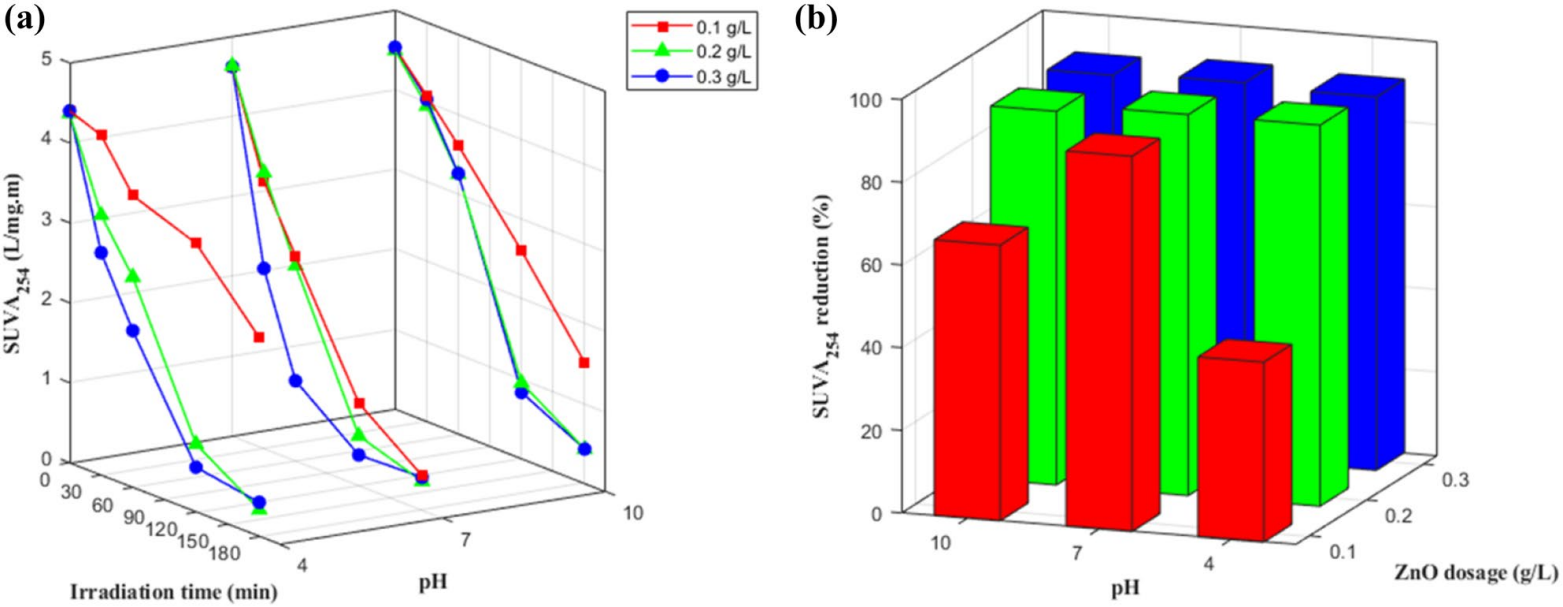
Changes of SUVA_254_ of DOM during photocatalysis under different ZnO dosages and pHs: (**a**) SUVA_254_ and (**b**) a total reduction (%).

Figure 3 presents the changes in SUVA_254_ during irradiation. Initially, the SUVA_254_ values were all higher than 4, ranging from 4.37 to 4.98, indicating that the organic matter was primarily composed of hydrophobic compounds with high molecular weights (HMWs)^9, 26^. There was a substantial reduction in SUVA_254_ (over 90% of initial values) after 180 min of irradiation in most of the samples except for two (pH 4 and pH 10 with 0.1 g/L ZnO). This was because of the preferential removal of aromatic chromophores over aliphatic moieties, followed by the transition of the DOM to non- or less-UV-absorbing substances^28^. This reduction in SUVA_254_ also indicates that HMW DOM was rapidly decomposed into organic compounds of lower molecular weight (LMW), which is supported by the lower DOC removal values compared to UV_254_-measured removal for the same reaction time^27^. A strong linear correlation (R^2^ = 0.92–0.98) was found between SUVA_254_ and DOC, and similar effects of ZnO dosage, pH level, and the presence of inorganic anions were observed.

### Change in EEMs

The changes in the EEMs of the DOM over 180 min of irradiation under optimal conditions (0.2 g/L ZnO and pH 7) are presented in Fig. S4. It was observed that the broad and strong peak at emission wavelengths above ~ 350 nm, commonly referred to as the humic-like peak, decreased significantly with increasing irradiation time. After 180 min, the fluorescence intensity for the measured wavelengths was almost zero, with no clear peaks.

The lower fluorescence intensity in the EEM plots of the DOM was likely due to the preferential photocatalytic degradation of the HMW fraction^27^, which led to an increase in the LMW fraction. This was supported by size-exclusion chromatography using DOC and UV_254_ detection, which also observed a reduction in fluorescence intensity with lower molecular weights based on the synchronous scan spectra of Aldrich HA fractions obtained with ultrafiltration after photocatalysis^27^. Moreover, the photocatalytic degradation of the HMW compounds in the DOM was similar to the previously reported photocatalytic degradation of NOM from a bog lake^29^. The photocatalytic degradation of DOM followed a similar sequence to other oxidation processes, such as the chlorination of NOM^30^ and the photocatalytic degradation of commercial HA using TiO_2_ and a solar UV-light simulator^27^.

### Behavior of the components during photocatalysis

#### EEM-PARAFAC components

Using 125 EEM samples from 25 experiments, two components (C1 and C2) were identified using PARAFAC modeling (Fig. S5 and Table S3). It was considered reasonable to extract two fluorophores from the samples because Sigma-Aldrich HA is known to be pedogenic with quite uniform sources^31^. C1 produced a maximum peak at an Ex/Em of 261 nm/ ≥ 500 nm, exhibiting a broad excitation spectrum and gradual emission above 350 nm, while C2 peaked at an Ex/Em of < 230 nm/438 nm, with a shoulder peak at the excitation wavelength range of 300–350 nm. Compared to C1, the location of the C2 peak is likely to have been blue-shifted in both the excitation and emission spectra, which would indicate that C1 has a higher molecular weight and more hydrophobic, rather than C2.

#### Behavior of the PARAFAC components

**Figure 4:**
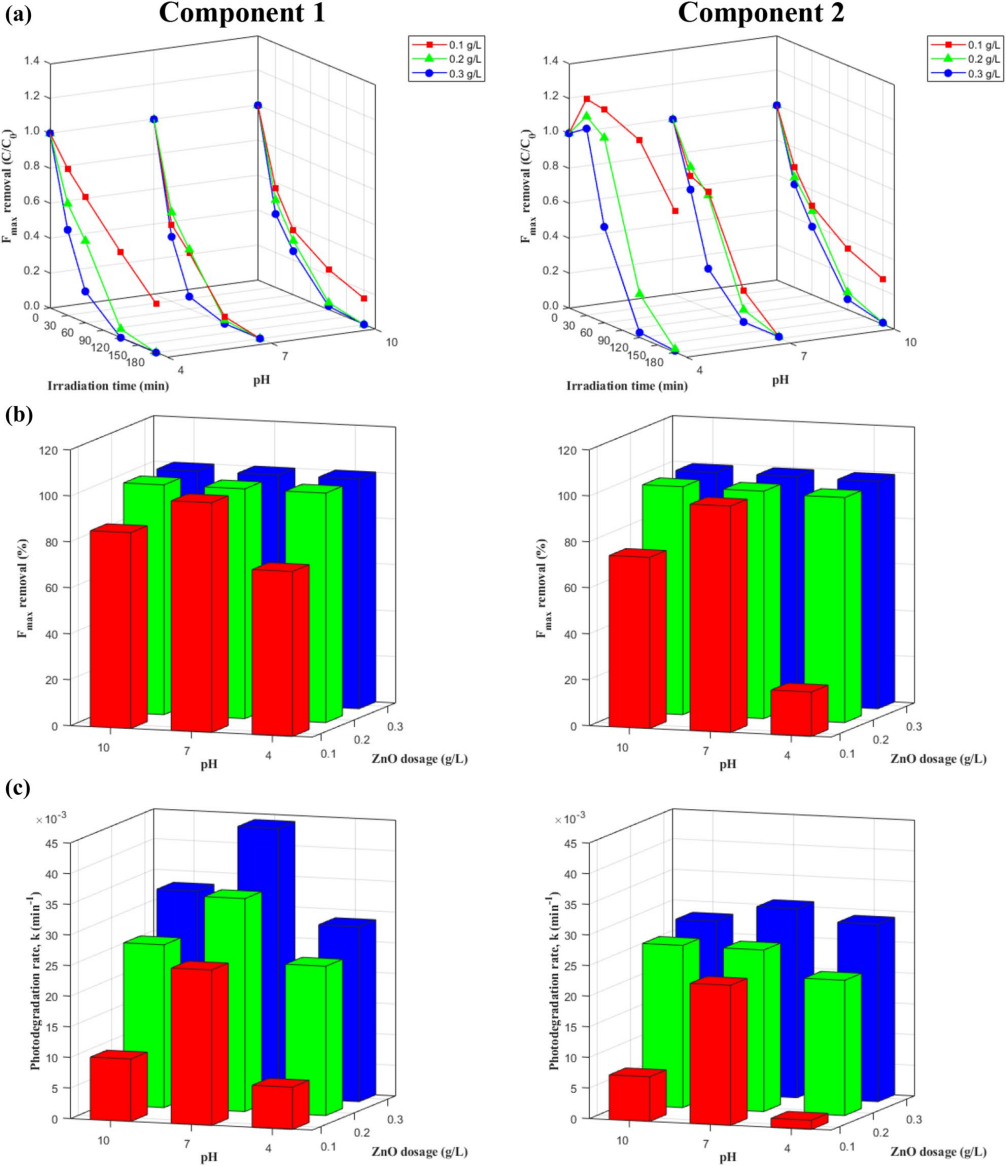
Changes of two EEM-PARAFAC components during photocatalysis under different ZnO dosages and pH values: (**a**) F_max_ degradation curves, (**b**) removal %, and (**c**) degradation rates.

**Figure 5:**
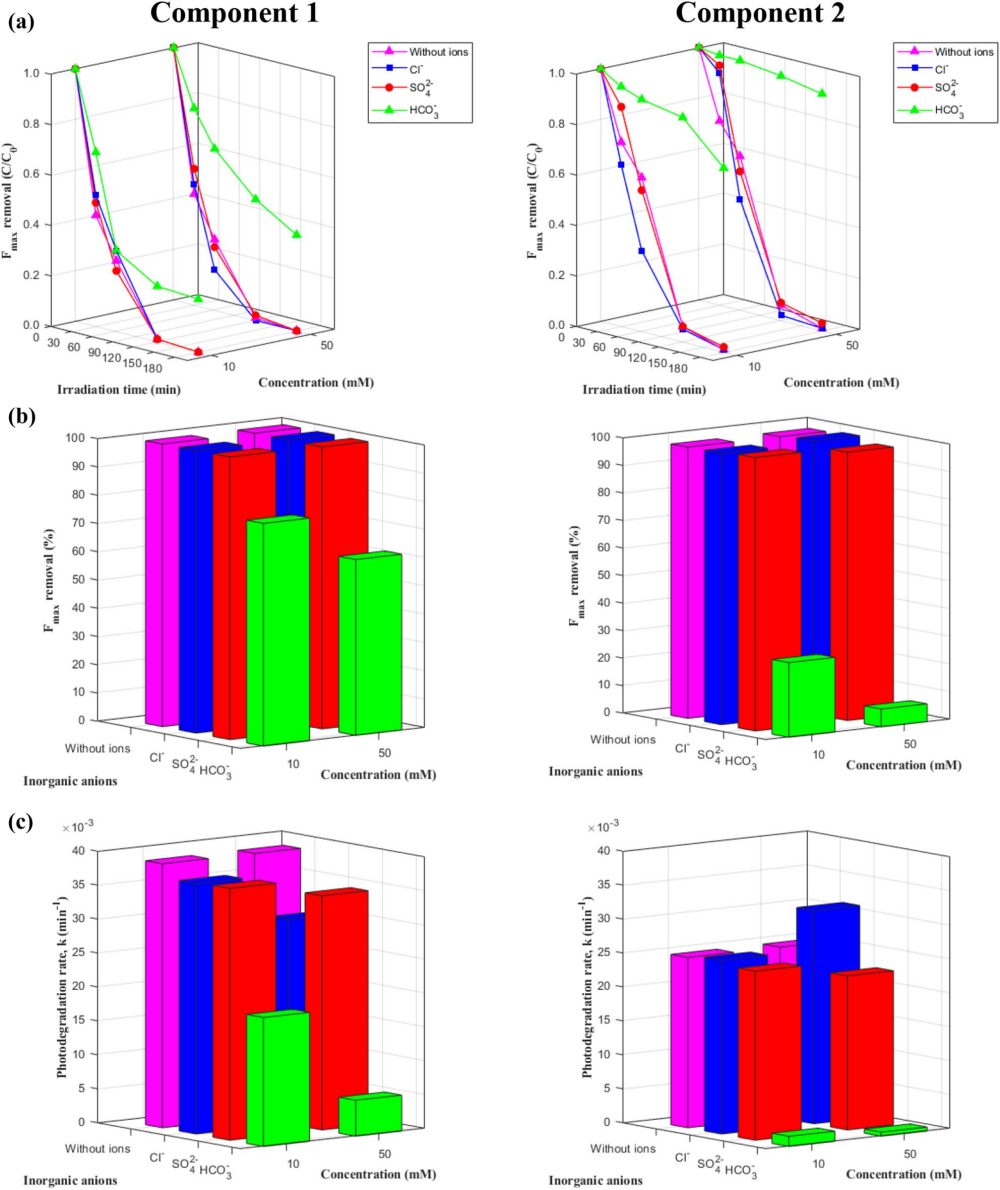
Effects of inorganic anions on degradation of two EEM-PARAFAC components during photocatalysis with 0.2 g/L ZnO at pH 7: (**a**) F_max_ degradation curves, (**b**) removal %, and (**c**) degradation rates.

The degradation of C1 and C2 based on F_max_ measurements is presented in Figs. 4 and 5 and Table S4. The degradation of both components fit a pseudo-first-order model (R^2^ = 0.94–1.00). The final percentage removal after 180 min was similar for the two components; however, the degradation rate was always faster for C1 than for C2 under the same experimental conditions over the first 120 min. In addition, the effects of ZnO dosage, pH, and the presence of inorganic anions on F_max_ removal and the photodegradation rate of C1 were similar to those described in the previous subsections. The highest F_max_ removal of C1 was 100% after 120 min of irradiation at a ZnO dosage of 0.3 g/L and a pH of 7, with no additional inorganic anions.

C2 was identified as terrestrial humic-like organic matter with shorter excitation and emission wavelengths than C1. Generally, its photodegradation rate fit a pseudo-first-order kinetic model (R^2^ = 0.93–1.00), with two exceptions at ZnO dosages of 0.1 g/L and 0.2 g/L and a pH of 4 (R^2^ = 0.36 and 0.86, respectively). It was observed that total F_max_ removal and the photodegradation rate increased with higher ZnO dosages, but there was no apparent relationship between pH level and degradation. Specifically, total removal and the photodegradation rate followed the order pH 7 > pH 10 > pH 4 with 0.1 g/L ZnO and the order pH 10 > pH 7 > pH 4 with 0.2 g/L ZnO. With a ZnO dosage of 0.3 g/L, total removal followed the order pH 10 > pH 7 > pH 4, while the photodegradation rate followed the order pH 7 > pH 4 > pH 10 (Fig. 4). A difference from previous results was also observed for the addition of inorganic anions. In the presence of inorganic anions, total F_max_ removal and the photodegradation rate followed the order Cl^−^ > no anions > SO_4_^2−^ > HCO_3_^−^. The highest total F_max_ removal was 99.36% after 180 min irradiation at a ZnO dosage of 0.3 g/L and a pH of 10, with no additional inorganic anions (Fig. 5).

Because the degradation behavior of both PARAFAC components followed a first-order exponential decay process, their photocatalytic degradation and kinetic rates could be directly compared. Total F_max_ removal and the photodegradation rate of C1 were higher than those of C2, which can be explained by the excitation and emission wavelengths of each component. Although both C1 and C2 were both identified as terrestrial humic-like organic matter, C1 represents a combination of peak A and peak C, exhibiting longer excitation and emission wavelengths than C2. With peaks at longer wavelengths, C1 may be associated with the structural condensation and polymerization of DOM^15, 32^. Indeed, more pronounced fluorescence at longer emission wavelengths in the EEMs of larger sized and/or more hydrophobic DOM fractions has been previously reported^33^. Therefore, the results indicate the preferential adsorption of more hydrophobic and larger DOM molecules onto minerals and/or nanoparticles, which has also been reported in previous studies^15, 34^. In addition, because C2 has shorter excitation wavelengths than C1, it would be less excited by visible light than C1.

**Figure 6:**
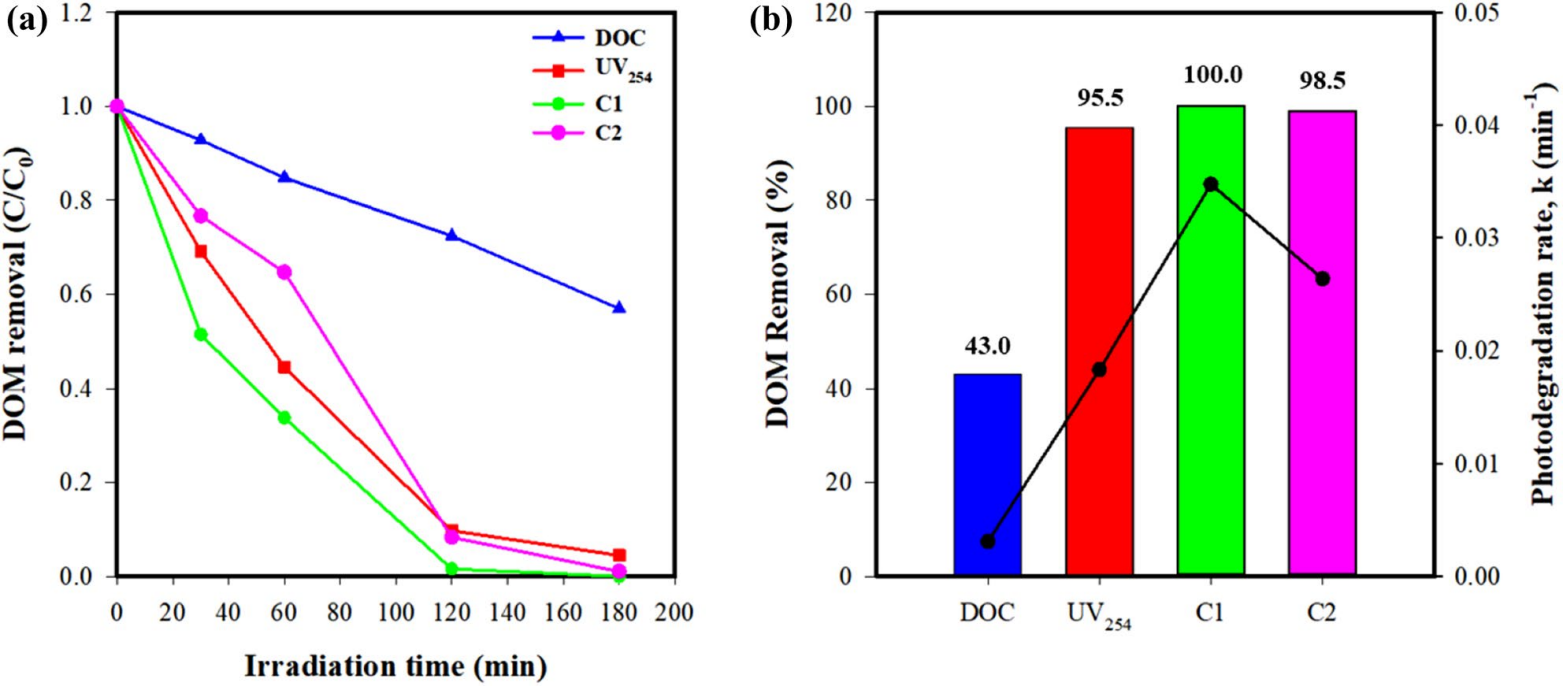
Changes in DOC, UV_254_, and two EEM-PARAFAC components during photocatalytic degradation of DOM with 0.2 g/L ZnO at pH 7 under artificial sunlight: (**a**) degradation curves and (**b**) removal % and degradation rates.

Total DOM removal and photodegradation rates calculated using DOC, UV_254_, and the PARAFAC components were also compared (Fig. 6). It was interesting to observe that the total removal and photodegradation rates calculated using the F_max_ of the two PARAFAC components were higher than those calculated using DOC and UV_254_. In particular, under optimal conditions (0.2 g/L ZnO, pH 7, and no inorganic anions), the total removal of C1 (100%) and C2 (98.97%) was observed to be higher than total UV_254_ removal (95.54%) and much higher than total DOC removal (43.04%), while the photodegradation rate of C1 was 11.27-fold and 8.55-fold higher than the photodegradation rates calculated with DOC and UV_254_, respectively. Similarly, the photodegradation rate of C2 was 1.90-fold and 1.44-fold higher than the photodegradation rates calculated with DOC and UV_254_, respectively. The more rapid degradation of fluorescence components compared to UV-absorbing moieties (i.e., UV_254_) could be explained by the fluorescence arising from the π*–π transitions in DOM molecules and its rapid extinction under UV irradiation^15, 16^.

The proposed reaction mechanism for the ZnO-assisted photocatalytic degradation of DOM under artificial sunlight is presented in Fig. S6. When ZnO is irradiated with artificial light containing photonic energy (hv), valence band hole (${h}_{VB}^{+}$) and conduction band electron (${e}_{CB}^{-}$) pairs are produced, as given in Eq. (10)^11^. The ${h}_{VB}^{+}$ reacts with H_2_O and hydroxide ions to yield ^·^OH (Eqs. 12 and 13)^7–9^. The reduction of dissolved or adsorbed O_2_ to ${O}_{2}^{\cdot-}$ by ${e}_{CB}^{\_}$ is depicted in Eq. (14)^7–9^. The ${O}_{2}^{\cdot-}$ is converted to H_2_O_2_ via disproportionation with protons (Eq. 15) or forms ${HO}_{2}^{\cdot}$ via protonation, which has a short lifetime due to the rapid reaction with ${O}_{2}^{\cdot-}$ or ${HO}_{2}^{\cdot}$ to form the more stable H_2_O_2_ (Eqs. 16 and 17)^7–9, 35^. The one-electron reduction of H_2_O_2_ produces ^·^OH (Eq. 19), while H_2_O_2_ can also react with ${O}_{2}^{\cdot-}$ to form ^·^OH (Eqs. 20 and 21)^7–9^. The generated ^·^OH is a powerful oxidizing agent that can attack DOM at or near the ZnO surface (Eq. 23). The reaction of ^·^OH with HAs (as a representative form of DOM) results in the release of LMW acids, amino acids, and ammonia^36^. The ${O}_{2}^{\cdot-}$ can also oxidize the DOM molecules (Eq. 24)^16^. Moreover, upon absorbing light, DOM can act as a photosensitizer in the generation of reactive species such as singlet oxygen (^1^O_2_), ^·^OH, and triplet DOM states (^3^DOM^*^), as given in Eqs. (25–28)^37^. ^3^DOM^*^ is a potent oxidant of many aquatic contaminants that react with target organic substances directly through electron and energy transfer mechanisms to generate reactive oxygen species such as ^1^O_2_, ^·^OH, and H_2_O_2_^37^, thus significantly influencing on the degradation of various fluorophores.

10 $$ZnO+hv \to {h}_{VB}^{+}+{e}_{CB}^{-}$$

11 $${H}_{2}O\to {OH}^{-}+{H}^{+}$$

12 $${\text{h}}_{{{\text{VB}}}}^{ + } + {\text{ H}}_{2} {\text{O}} \to { }^{ \cdot } {\text{OH }} + {\text{ H}}^{ + }$$

13 $${\text{h}}_{{{\text{VB}}}}^{ + } + {\text{ OH}}^{ - } \to { }^{ \cdot } {\text{OH}}$$

14 $${\text{e}}_{{{\text{CB}}}}^{\_} + {\text{ O}}_{2} { } \to {\text{ O}}_{2}^{ \cdot - }$$

15 $${\text{O}}_{2}^{ \cdot - } + 2{\text{H}}^{ + } + {\text{e}}_{{{\text{CB}}}}^{\_} { } \to {\text{H}}_{2} {\text{O}}_{2}$$

16 $${\text{O}}_{2}^{ \cdot - } + {\text{H}}^{ + } \to {\text{HO}}_{2}^{ \cdot } \;\;\;\;\;\;\left( {k = 2.1 \times 10^{10} {\text{ M}}^{ - 1} {\text{s}}^{ - 1} } \right)$$

17 $${\text{HO}}_{2}^{ \cdot } + {\text{HO}}_{2}^{ \cdot } \to {\text{H}}_{2} {\text{O}}_{2} + {\text{O}}_{2} \;\;\;\;\;\;\;\;\left( {k = 8.3 \times 10^{5} {\text{ M}}^{ - 1} {\text{s}}^{ - 1} } \right)$$

18 $$^{ \cdot } {\text{OH }} +^{ \cdot } {\text{OH}} \to {\text{H}}_{2} {\text{O}}_{2} \;\;\;\;\;\;\;\;\;\;\;\;\left( {k = 5.5 \times 10^{9} {\text{ M}}^{ - 1} {\text{s}}^{ - 1} } \right)$$

19 $${\text{H}}_{2} {\text{O}}_{2} + {\text{H}}^{ + } + {\text{e}}_{{{\text{CB}}}}^{\_} \to { }^{ \cdot } {\text{OH }} + {\text{ H}}_{{2}} {\text{O}}$$

20 $${\text{H}}_{2} {\text{O}}_{2} + {\text{O}}_{2}^{ \cdot - } \to {\text{OH}}^{ - } + {\text{O}}_{2} + ^{ \cdot } {\text{OH}} \;\;\;\;\;\;\;\left( {k = 0.13{\text{ M}}^{ - 1} {\text{s}}^{ - 1} } \right)$$

21 $${\text{H}}_{2} {\text{O}}_{2} + {\text{hv}} \to {2}^{ \cdot } {\text{OH}}$$

22 $${\text{H}}_{2} {\text{O}}_{2} +^{ \cdot } {\text{OH}} \to {\text{H}}_{{2}} {\text{O}}\;\;\;\;\;\;\;\left( {k = 2.7 \times 10^{7} {\text{ M}}^{ - 1} {\text{s}}^{ - 1} } \right)$$

23 $${\text{DOM }} +^{ \cdot } {\text{OH}} \to {\text{CO}}_{{2}} + {\text{ H}}_{{2}} {\text{O }} + {\text{ Products}}\;\;\;\;\;\;\;\;\left( {k = 1.7 \times 10^{8} {\text{ Mc}}^{ - 1} {\text{s}}^{ - 1} } \right)$$

24 $${\text{DOM }} + {\text{O}}_{2}^{ \cdot - } \to {\text{CO}}_{2} + {\text{ H}}_{{2}} {\text{O }} + {\text{ Products}}$$

25 $${\text{DOM }} + {\text{ hv}} \to^{{1}} {\text{DOM}}^{*} \to^{{3}} {\text{DOM}}^{*}$$

26 $$^{{3}} {\text{DOM}}^{*} + {\text{ O}}_{{2}} \to^{{1}} {\text{DOM }} +^{{1}} {\text{O}}_{{2}}$$

27 $${\text{DOM}} + {\text{ O}}_{2}^{ \cdot - } \to {\text{DOM}}^{ \cdot - } + {\text{O}}_{2}$$

28 $$2{\text{O}}_{2}^{ \cdot - } + 2{\text{H}}^{ + } { } \to {\text{ H}}_{2} {\text{O}}_{2} + {\text{ O}}_{2} \;\;\;\;\;\;\;\;\;\;\left( {k = 4.0 \times 10^{4} {\text{ M}}^{ - 1} {\text{s}}^{ - 1} } \right)$$

**Figure 7:**
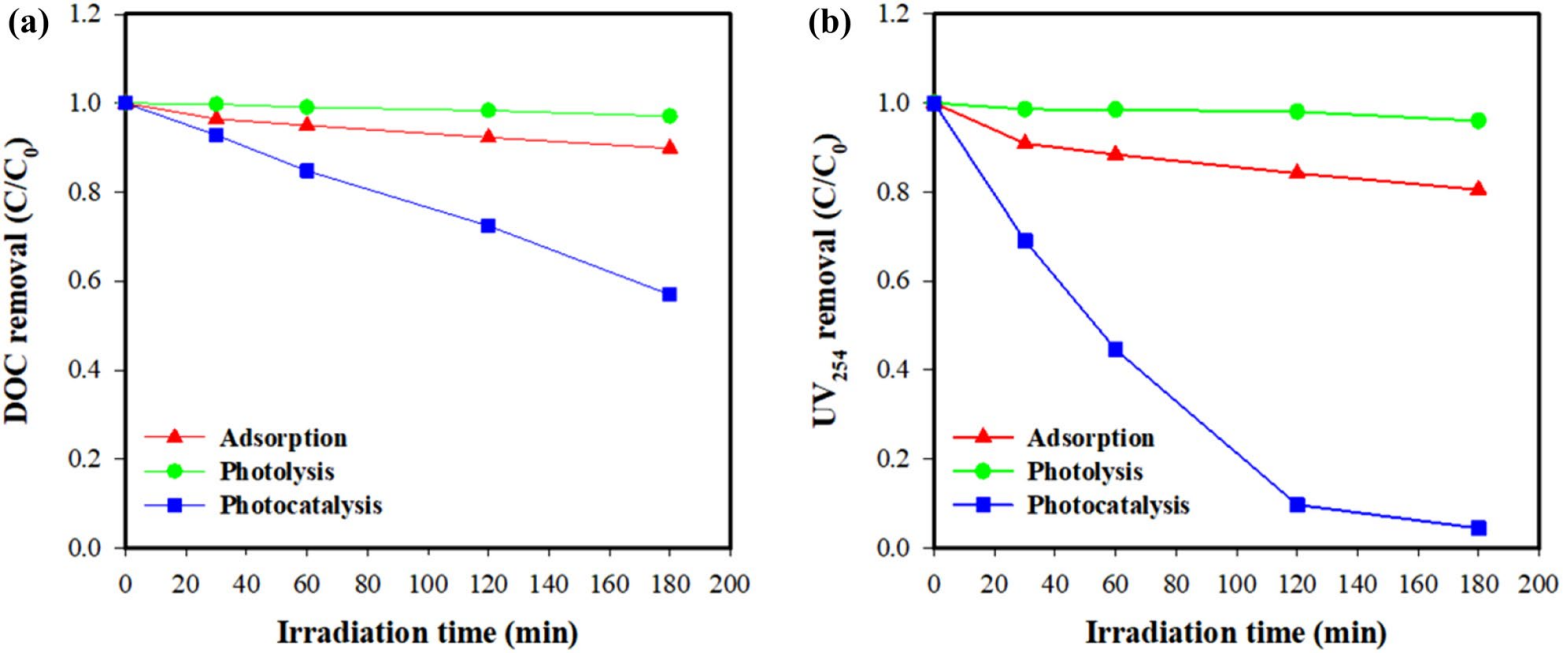
Comparison of photolysis, photocatalysis and adsorption of DOM with 0.2 g/L ZnO at pH 7: (**a**) DOC concentrations and (**b**) UV_254_ values.

The effects of photolysis, adsorption, and photocatalysis on the degradation of DOM was also assessed. Figure 7 compares the results for total removal and the degradation rate for these three processes. The total DOM removal and degradation rate calculated with DOC are illustrated in Fig. 7a. After 180-min irradiation, total DOM removal was 2.92% for photolysis, 10.15% for adsorption, and 43.04% for photocatalysis. The photocatalytic rate was 18.6-fold and 5.5-fold higher than that for photolysis and adsorption. The DOM removal and degradation rate calculated with UV_254_ (Fig. 7b) also revealed that photocatalysis was more effective than the other two processes. Specifically, total DOM removal was 4.03% for photolysis, 19.46% for adsorption, and 95.45% for photocatalysis, while the degradation rate was 93.6-fold and 17.0-fold higher than that of photolysis and adsorption, respectively. Based on these results, we can conclude that adsorption by ZnO only and photolysis only play a minor role in DOM removal, while the synergistic effects of photocatalysis are vital to this process.

## Conclusions

The photocatalytic degradation of DOM under artificial sunlight irradiation using ZnO as a photocatalyst was examined under various experimental settings (i.e., ZnO dosage, pH level, and the presence of inorganic anions such as Cl^−^, SO_4_^2−^, and HCO_3_^−^). Changes in the DOM were analyzed using UV_254_, SUVA_254_, DOC, and fluorescence EEM-PARAFAC analysis. The following conclusions can be drawn from the results:

- The photocatalytic degradation of DOM followed a pseudo-first-order kinetic model. Adsorption by ZnO only and photolysis had little impact on DOM degradation, with photocatalysis playing the dominant role.
- The EEM-PARAFAC approach decomposed fluorescent DOM into two types of terrestrial humic-like organic matter (C1 and C2).
- Similar trends in total DOM removal and photodegradation rates were observed when calculated using DOC, UV_254_, and the F_max_ of C1. The total removal and degradation rates increased as ZnO dosage increased and were highest in neutral conditions (pH 7) and lowest in acidic conditions (pH 4). The presence of inorganic anions inhibited the photocatalytic degradation of DOM, with the strongest inhibition effect observed when HCO_3_^−^ was added to the solution.
- Measurements taken using PARAFAC component C2 exhibited a similar relationship with ZnO dosage to that shown by DOC, UV_254_, and the F_max_ of C1. However, pH level did not appear to have a consistent effect on degradation. In the presence of inorganic anions, a different trend was noted. The addition of Cl^−^ improved rather than inhibited removal efficiency, while total F_max_ removal and the photodegradation rate followed the order Cl^−^ > no anions > SO_4_^2−^ > HCO_3_^−^.
- A comparison of the total DOM removal and photodegradation rates calculated using DOC, UV_254_, and the PARAFAC components was made. The total removal and photodegradation rates calculated using the F_max_ value of the two PARAFAC components were higher than those calculated using DOC and UV_254_.

## Supplementary information

Supplementary Information.
