# Supplementary material for: Photocatalytic degradation of dissolved organic matter under ZnO-catalyzed artificial sunlight irradiation system
Source: Sci Rep. 2020 Aug 4;10:13090. doi: 10.1038/s41598-020-69115-7 (PMC7403426; doi:10.1038/s41598-020-69115-7)
Supplement: Supplementary file 1 — Supplementary Information. [file 41598_2020_69115_MOESM1_ESM.docx]

**Supplementary information**

**Photocatalytic Degradation of Dissolved Organic Matter Under ZnO-catalyzed Artificial Sunlight Irradiation System**

**Thao Thi Nguyen, Seong-Nam Nam*, Jungryul Kim, Jeill Oh**

Department of Civil and Environmental Engineering, Chung-Ang University, 84 Heukseok-ro, Dongjak-gu, Seoul, 06974, Republic of Korea

*Co-corresponding authors. E-mail: [namsn76@gmail.com](mailto:namsn76@gmail.com)

**List of Figures**


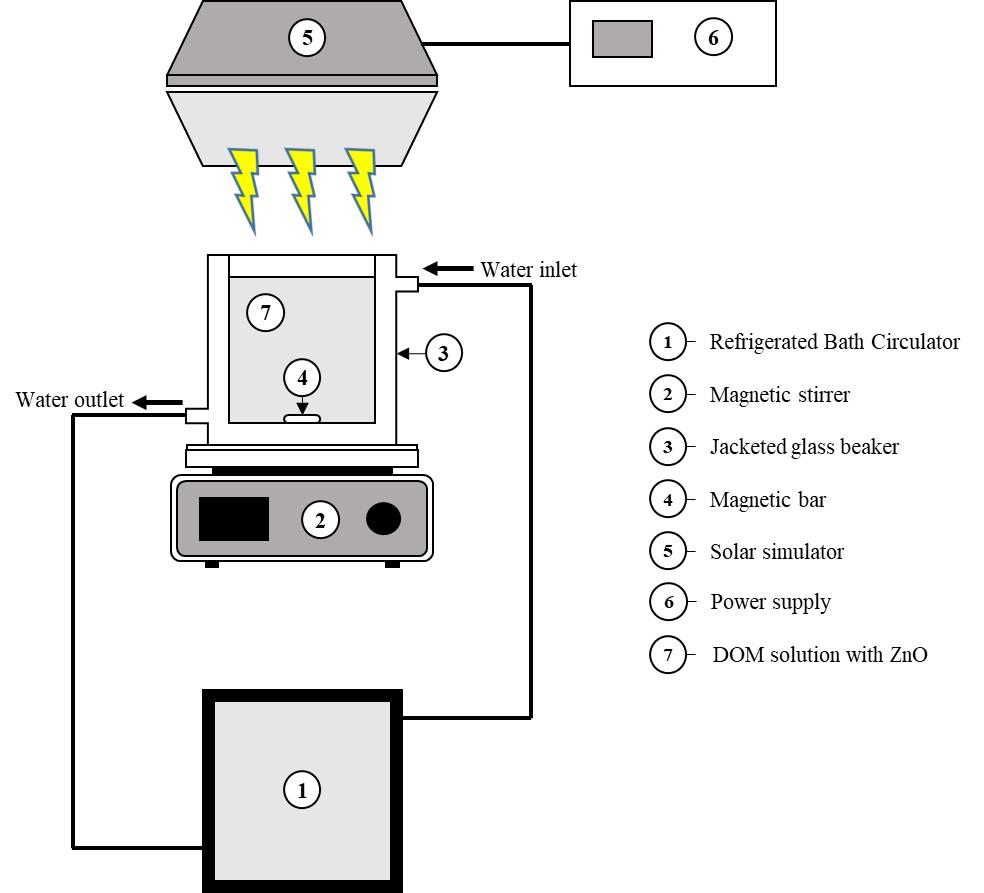


**Figure S1**. Experimental setup for the photocatalytic degradation of DOM by ZnO under artificial sunlight.

**
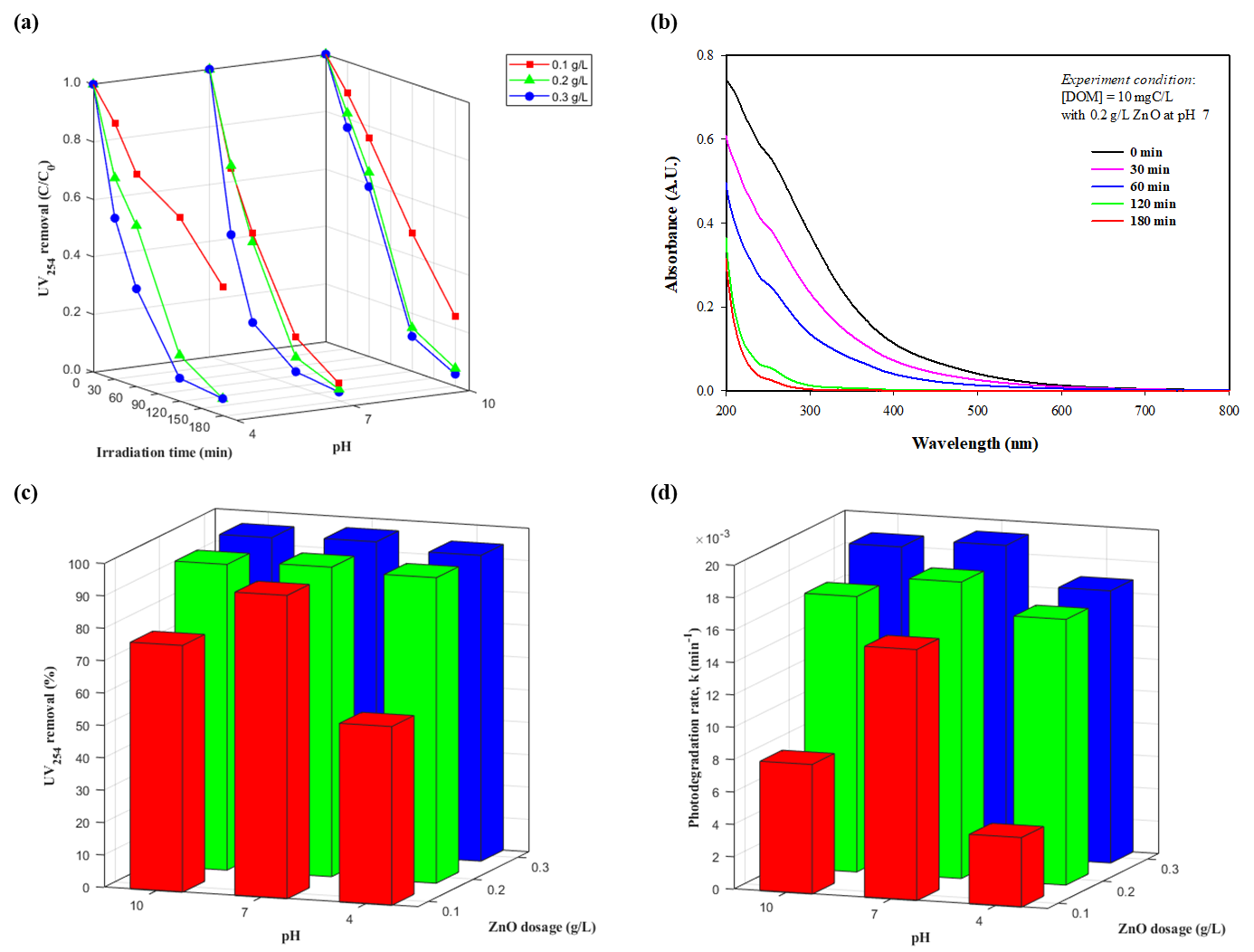
**

**Figure S2.** Effect of ZnO dosage and pH on UV_254_ removal for photocatalytic degradation of DOM: (a) degradation curves, (b) UV/Vis spectra changes during the DOM degradation with 0.2 g/L ZnO at pH 7, (c) removal %, and (d) degradation rates.

**
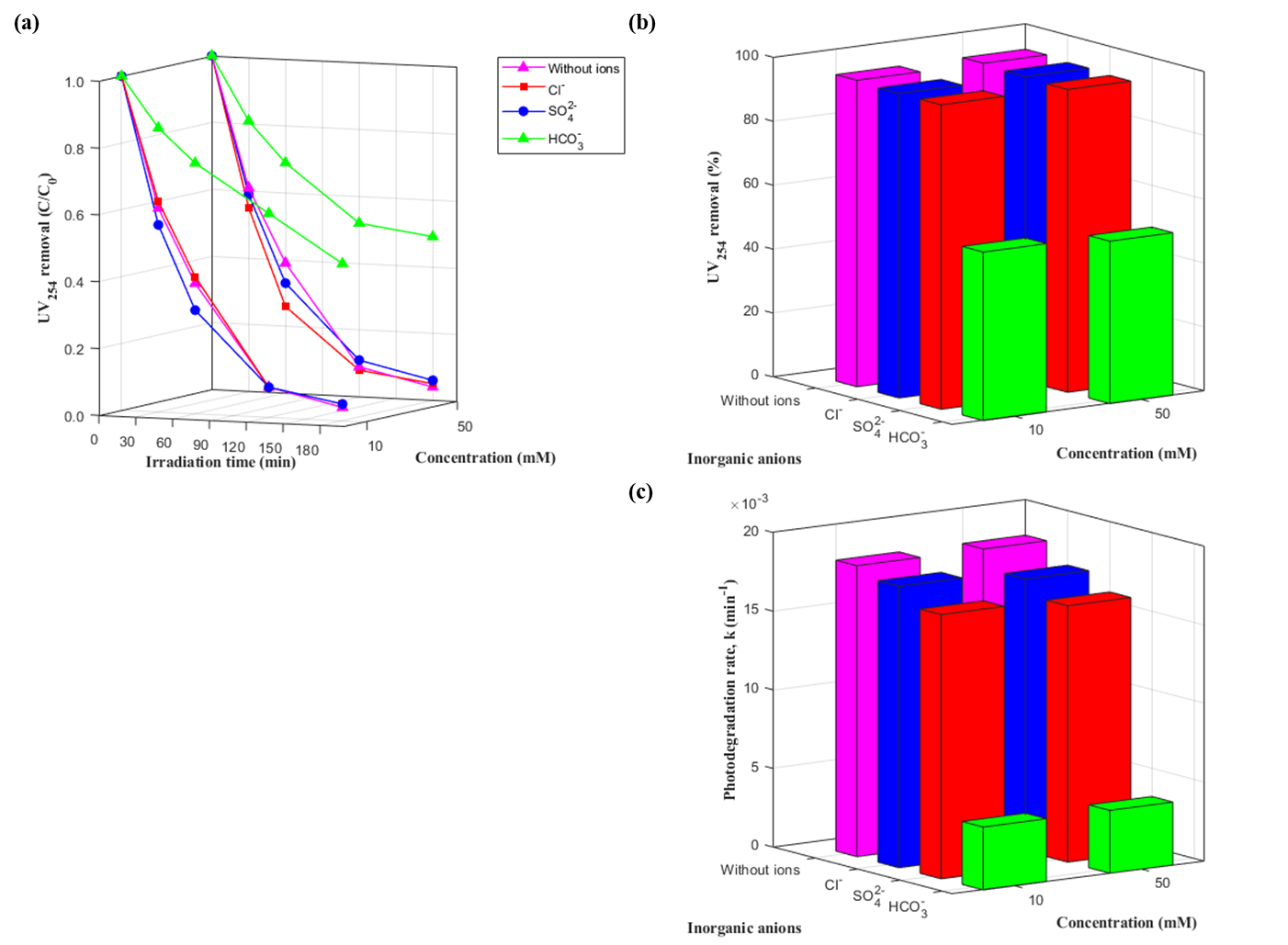
**

**Figure S3.** Effect of inorganic anions on UV_254_ removal for photocatalytic degradation of DOM with 0.2 g/L ZnO at pH7: (a) degradation curves, (b) removal %, and (c) degradation rates.


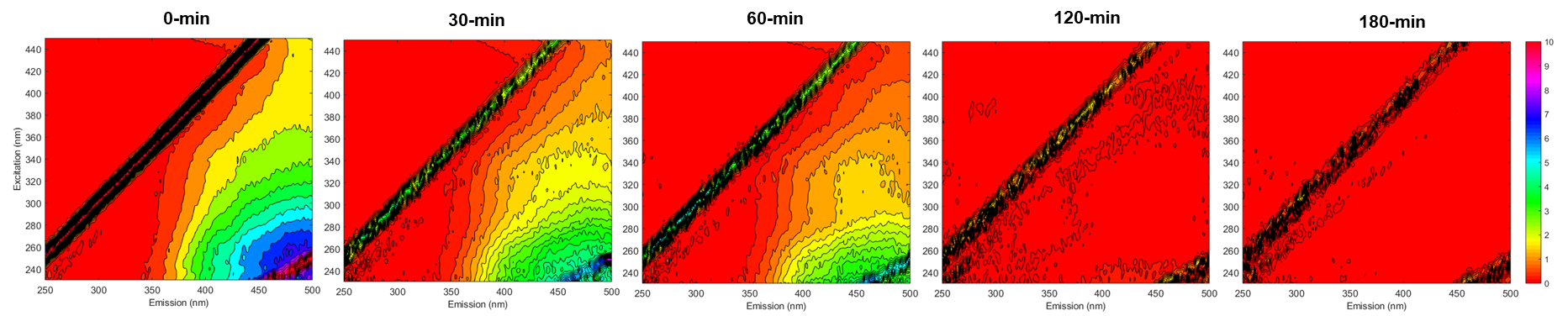


**Figure S4**. Changes of EEM of DOM by photocatalysis with 0.2 g/L ZnO at pH 7 for the 180-min reaction; 0-min ~ 180-min denote duration of photocatalysis under the artificial sunlight irradiation.


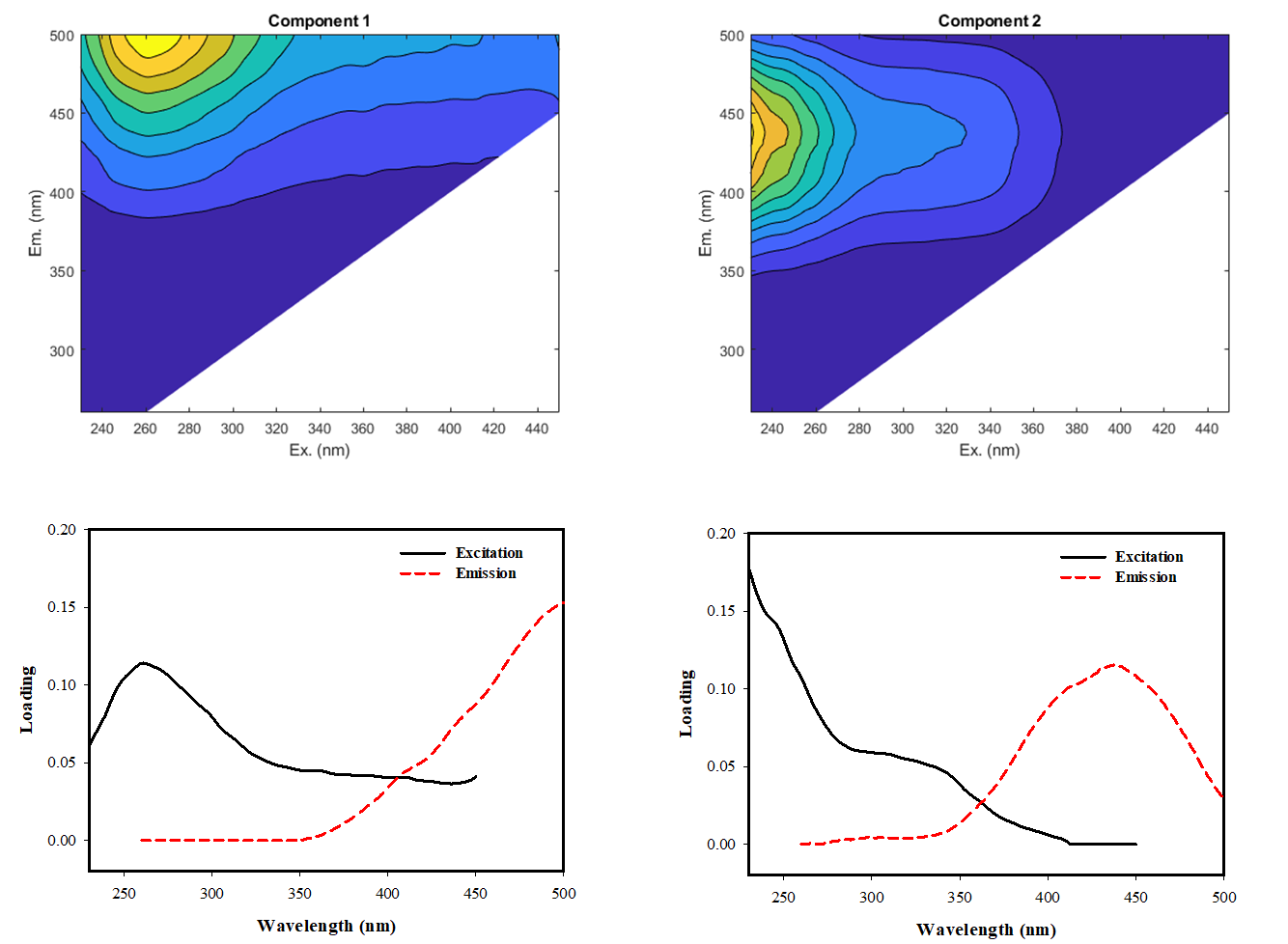


**Figure S5**. PARAFAC model output; (top row) contours of the two fluorescent components and (bottom row) the corresponding excitation/emission loadings.


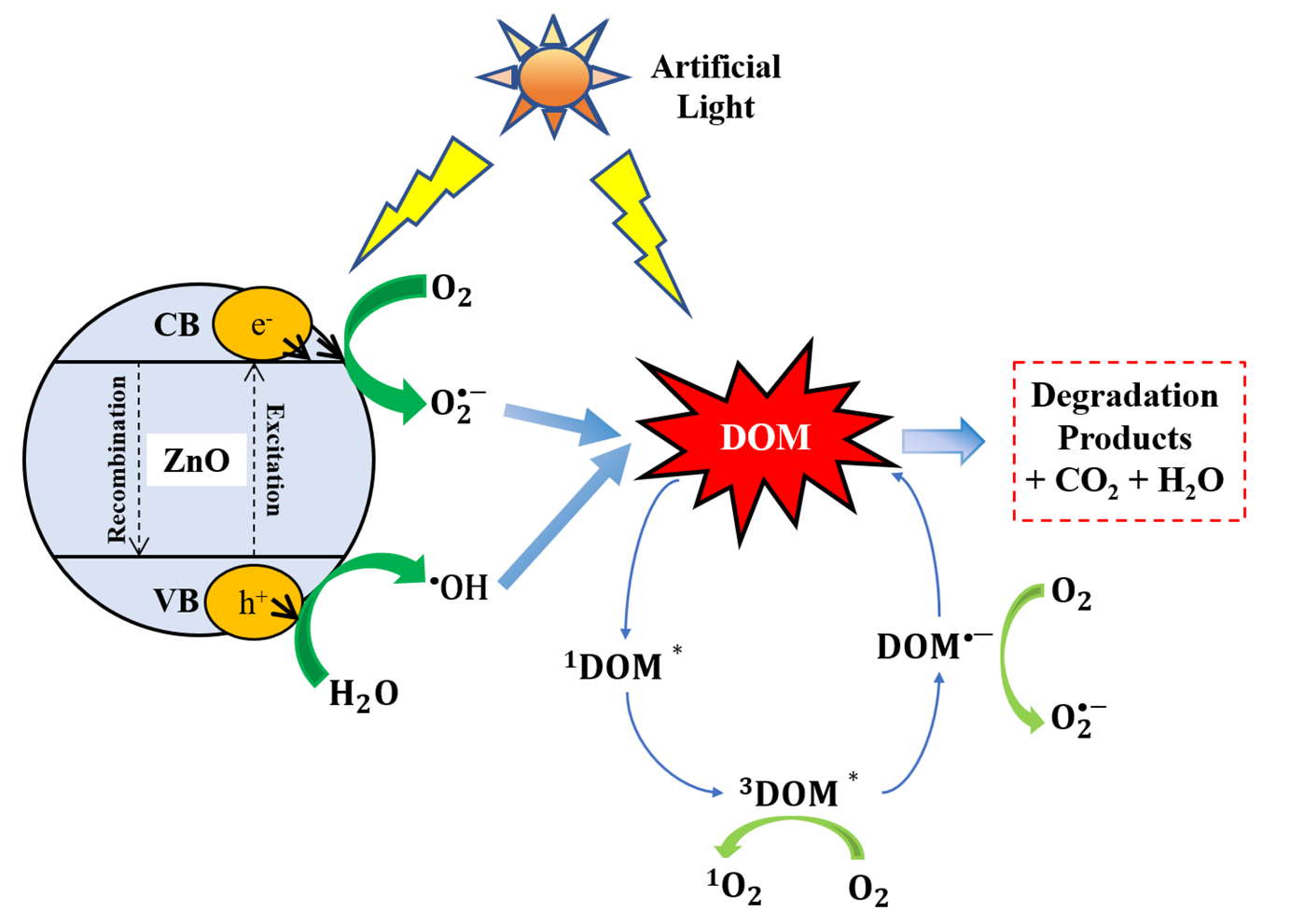


**Figure S6**. Proposed mechanisms for the photocatalytic degradation of DOM by ZnO under artificial sunlight.

**List of Tables**

**Table S1**. Changes in DOC, UV_254_, and two EEM-PARAFAC components during photocatalytic degradation of DOM with different pH values, ZnO dosages, and inorganic anions.

| **No.** | **Experimental conditions** | | | **Removal**  **(%)** | | **Photodegradation rate, k**  **(min ^-1^)** | |
| --- | --- | --- | --- | --- | --- | --- | --- |
|  | **pH** | **ZnO dosage (g/L)** | **Anions**  **(mM)** | **DOC** | **UV_254_** | **DOC** | **UV_254_** |
| 1 | 4 | 0.1 | without | 20.64 | 55.20 | 0.0012 | 0.0043 |
| 2 | 4 | 0.2 | without | 27.23 | 94.32 | 0.0017 | 0.0164 |
| 3 | 4 | 0.3 | without | 42.06 | 94.39 | 0.0030 | 0.0168 |
| 4 | 7 | 0.1 | without | 31.24 | 93.65 | 0.0021 | 0.0155 |
| 5 | 7 | 0.2 | without | 43.04 | 95.54 | 0.0031 | 0.0183 |
| 6 | 7 | 0.3 | without | 60.88 | 96.54 | 0.0052 | 0.0192 |
| 7 | 10 | 0.1 | without | 28.50 | 76.19 | 0.0019 | 0.0080 |
| 8 | 10 | 0.2 | without | 41.50 | 94.37 | 0.0030 | 0.0170 |
| 9 | 10 | 0.3 | without | 56.11 | 95.79 | 0.0044 | 0.0187 |
| 10 | 7 | 0.2 | without | 43.85 | 95.87 | 0.0031 | 0.0185 |
| 11 | 7 | 0.2 | [$\mathrm{Cl}^{─}$] = 10mM | 40.05 | 95.25 | 0.0028 | 0.0179 |
| 12 | 7 | 0.2 | [$\mathrm{Cl}^{─}$] = 50mM | 34.58 | 95.24 | 0.0025 | 0.0173 |
| 13 | 7 | 0.2 | [$\mathrm{SO}_{4}^{2─}$] = 10mM | 37.95 | 94.72 | 0.0025 | 0.0168 |
| 14 | 7 | 0.2 | [$\mathrm{SO}_{4}^{2─}$] = 50mM | 34.42 | 94.25 | 0.0024 | 0.0163 |
| 15 | 7 | 0.2 | [$\mathrm{HCO}_{3}^{─}$] = 10mM | 22.78 | 52.71 | 0.0015 | 0.0040 |
| 16 | 7 | 0.2 | [$\mathrm{HCO}_{3}^{─}$] = 50mM | 16.76 | 50.82 | 0.0010 | 0.0040 |

**Table S2:** Reported reaction kinetics and rate constants of Cl^─^, HCO_3_^─^, and SO_4_^2─^ anions

| **No.** | **Reaction** | **Rate Constant (M^-1^s^-1^)** | **Reference** |
| --- | --- | --- | --- |
| **Chloride reactions** | | | |
| 1 | $\mathrm{Cl}^{─}+h_{\mathrm{VB}}^{+}\to\mathrm{Cl}^{\bullet}$ |  | ^1^ |
| 2 | $\mathrm{Cl}^{─}+$^•^OH $\to\mathrm{HOCl}^{\bullet─}$ | 4.3 x 10^9^ | ^2^ |
| 3 | $\mathrm{HOCl}^{\bullet─}\to\mathrm{Cl}^{─}+$^•^OH | 6.1 x 10^9^ | ^2^ |
| 4 | $\mathrm{HOCl}^{\bullet─}+ H^{+}\to\mathrm{Cl}^{\bullet}+H_{2}O$ | 2.1 x 10^10^ | ^2^ |
| 5 | $\mathrm{Cl}^{\bullet}+H_{2}O\to\mathrm{HOCl}^{\bullet─}+H^{+}$ | 2.5 x 10^5^ | ^2^ |
| 6 | $\mathrm{HOCl}^{\bullet─}+ \mathrm{Cl}^{─}\to\mathrm{Cl}_{2}^{\bullet─}+\mathrm{OH}^{─}$ | 1.0 x 10^4^ | ^3^ |
| 7 | $\mathrm{Cl}^{\bullet}+\mathrm{Cl}^{─}\to\mathrm{Cl}_{2}^{\bullet─}$ | 8.5 x 10^9^ | ^4^ |
| 8 | $\mathrm{Cl}_{2}^{\bullet─}\to\mathrm{Cl}^{\bullet}+\mathrm{Cl}^{─}$ | 6.0 x 10^4^ | ^4^ |
| 9 | $\mathrm{Cl}_{2}^{\bullet─}+$^•^OH $\to HOCl +\mathrm{Cl}^{─}$ | 1.0 x 10^9^ | ^5^ |
| 10 | $\mathrm{Cl}_{2}^{\bullet─}+\mathrm{Cl}_{2}^{\bullet─}\to\mathrm{Cl}_{2}+2\mathrm{Cl}^{-}$ | 9.0 x 10^9^ | ^4^ |
| 11 | $\mathrm{Cl}_{2}^{\bullet─}+\mathrm{Cl}^{\bullet}\to\mathrm{Cl}_{2}+\mathrm{Cl}^{-}$ | 2.1 x 10^9^ | ^4^ |
| 12 | $\mathrm{Cl}_{2}^{\bullet─}+O_{2}^{\bullet─}\to O_{2}+2\mathrm{Cl}^{-}$ | 2.0 x 10^9^ | ^6^ |
| 13 | $\mathrm{Cl}_{2}^{\bullet─}+H_{2}O\to HClOH+\mathrm{Cl}^{-}$ | 1.3 x 10^3^ | ^7^ |
| 14 | $HClOH\to\mathrm{HOCl}^{\bullet─}+H^{+}$ | 1.0 x 10^8^ | ^7^ |
| 15 | $HClOH\to\mathrm{Cl}^{\bullet}+H_{2}O$ | 1.0 x 10^2^ | ^7^ |
| 16 | $HClOH+\mathrm{Cl}^{─}\to\mathrm{Cl}_{2}^{\bullet─}+H_{2}O$ | 5.0 x 10^9^ | ^7^ |
| 17 | $\mathrm{Cl}^{\bullet}+HOCl\to\mathrm{ClO}^{\bullet}+H^{+}+\mathrm{Cl}^{-}$ | 3.0 x 10^9^ | ^8^ |
| 18 | $\mathrm{Cl}^{\bullet}+\mathrm{Cl}^{\bullet}\to\mathrm{Cl}_{2}$ | 8.8 x 10^7^ | ^9^ |
| 19 | $\mathrm{Cl}_{2}+\mathrm{Cl}^{─}\to\mathrm{Cl}_{3}^{─}$ | 2.0 x 10^4^ | ^10^ |
| 20 | $\mathrm{Cl}_{3}^{─}\to\mathrm{Cl}_{2}+\mathrm{Cl}^{─}$ | 1.1 x 10^5^ | ^10^ |
| 21 | $\mathrm{Cl}_{3}^{─}+O_{2}^{\bullet─}\to\mathrm{Cl}_{2}^{\bullet─}+\mathrm{Cl}^{-}+O_{2}$ | 3.8 x 10^9^ | ^6^ |
| 22 | $\mathrm{Cl}_{2}+H_{2}O\to HOCl +\mathrm{Cl}^{─}+H^{+}$ | 15 | ^11^ |
| 23 | $\mathrm{Cl}_{2}+O_{2}^{\bullet─}\to O_{2}+\mathrm{Cl}_{2}^{\bullet─}$ | 1.0 x 10^9^ | ^6^ |
| **Bicarbonate reactions** | | | |
| 24 | $\mathrm{HCO}_{3}^{─}+ h_{\mathrm{VB}}^{+}\to\mathrm{CO}_{3}^{\bullet─}+ H_{2}O$ |  | ^12^ |
| 25 | $\mathrm{HCO}_{3}^{─}$ + ^•^OH $\to\mathrm{CO}_{3}^{\bullet─}+ H_{2}O$ | 8.5 x 10^6^ | ^13^ |
| 26 | $\mathrm{CO}_{3}^{\bullet─}+$^•^OH $\to$ Product | 3.0 x 10^9^ | ^14^ |
| 27 | $\mathrm{CO}_{3}^{\bullet─}+O_{2}^{\bullet─}\to\mathrm{CO}_{3}^{2─}+O_{2}$ | 6.0 x 10^8^ | ^14^ |
| 28 | $\mathrm{CO}_{3}^{\bullet─}+\mathrm{CO}_{3}^{\bullet─}\to$ Product | 3.0 x 10^7^ | ^14^ |
| **Sulfate reactions** | | | |
| 29 | $\mathrm{SO}_{4}^{2─}+ h_{\mathrm{VB}}^{+} \to\mathrm{SO}_{4}^{\bullet─}$ |  | ^1^ |
| 30 | $\mathrm{SO}_{4}^{\bullet─}+ e_{\mathrm{CB}}^{─} \to\mathrm{SO}_{4}^{2─}$ |  | ^15^ |
| 31 | $\mathrm{SO}_{4}^{2─}$ + ^•^OH $\to\mathrm{SO}_{4}^{\bullet─}+ \mathrm{OH}^{─}$ | 1.18 x 10^6^ | ^16^ |
| 32 | $\mathrm{SO}_{4}^{\bullet─}+$^•^OH $\to\mathrm{HSO}_{5}^{─}$ | 1.0 x 10^10^ | ^17^ |
| 33 | $\mathrm{SO}_{4}^{\bullet─}+H_{2}O\to\mathrm{HSO}_{4}^{─}+$^•^OH | 660 | ^18^ |
| 34 | $\mathrm{SO}_{4}^{\bullet─}+\mathrm{HSO}_{5}^{─}\to\mathrm{SO}_{5}^{\bullet─}+\mathrm{SO}_{4}^{2─}$ | 1.0 x 10^6^ | ^17^ |
| 35 | $\mathrm{SO}_{5}^{\bullet─}+\mathrm{SO}_{5}^{\bullet─}\to{2SO}_{4}^{\bullet─}+O_{2}$ | 2.1 x 10^8^ | ^17^ |
| 36 | $\mathrm{SO}_{5}^{\bullet─}+\mathrm{SO}_{5}^{\bullet─}\to S_{2}O_{8}^{2─}+O_{2}$ | 2.2 x 10^8^ | ^17^ |
| 37 | $\mathrm{SO}_{4}^{\bullet─}+\mathrm{SO}_{4}^{\bullet─}\to S_{2}O_{8}^{2─}$ | 7.0 x 10^8^ | ^17^ |
| 38 | $\mathrm{SO}_{4}^{\bullet─}+S_{2}O_{8}^{2─}\to\mathrm{SO}_{4}^{2─}+S_{2}O_{8}^{\bullet─}$ | 6.3 x 10^5^ | ^17^ |
| 39 | $S_{2}O_{8}^{2─}+$ ^•^OH $\to S_{2}O_{8}^{\bullet─}$+$\mathrm{OH}^{─}$ | 1.2 x 10^7^ | ^17^ |
| 40 | $\mathrm{HSO}_{5}^{─}+$^•^OH $\to\mathrm{SO}_{5}^{\bullet─}+H_{2}O$ | 5.0 x 10^6^ | ^17^ |
| 41 | $\mathrm{SO}_{4}^{\bullet─}+O_{2}^{\bullet─}\to\mathrm{SO}_{4}^{2─}+O_{2}$ | 6.0 x 10^9^ | ^17^ |
| 42 | $\mathrm{SO}_{4}^{2─}+ H^{+} \leftrightarrow\mathrm{HSO}_{4}^{─}$ | 1.2 x 10^-2^ | ^19^ |
| 43 | $\mathrm{HSO}_{4}^{─}+$ ^•^OH $\to\mathrm{SO}_{4}^{\bullet─}+H_{2}O$ | 6.9 x 10^5^ | ^19^ |
| 44 | $S_{2}O_{8}^{2─}+e_{\mathrm{CB}}^{─} \to\mathrm{SO}_{4}^{\bullet─}+\mathrm{SO}_{4}^{2─}$ | 1.2 x 10^10^ | ^20^ |

**Table S3**. Spectral characteristics of the two fluorescent components identified by PARAFAC in this study and the comparison with those previously identified. The maxima wavelengths are presented in excitation/emission wavelengths.

| **Components** | **Ex_max_/Em_max_** | **Traditional peak** ^21^ | **Comparison with other studies using PARAFAC** | **Description and probable sources** | **Reference** |
| --- | --- | --- | --- | --- | --- |
| C1 | 261/≥500 | Peak A: 230-260/380-460  Peak C: 320–360/420–480 | C3: 260(370)/490 | Terrestrial humic substances, widespread "A" and "C" peaks | ^21-22^ |
|  |  |  | P3: < 260(380)/498 | Terrestrial humic substances, widespread "A" and "C" peaks | ^23^ |
|  |  |  | C1: 260(360)/480 | Terrestrial humic substances | ^24^ |
|  |  |  | C2: 260-300(350-400)/420-520 | Terrestrial humic substances "A" and "C" peaks | ^25^ |
|  |  |  | C2: <240-275(339-420)/434-520 | Humic-like | ^26^ |
|  |  |  | C4: 265(370)/490 | Terrestrial humic-like substances | ^27^ |
|  |  |  | C4: 250 (360)/500 | Humic-like | ^28^ |
|  |  |  | C3: 272(382)/504 | Terrestrial humic-like | ^29^ |
| C2 | <230/438 | Peak A: 230-260/380-460 | C1: <240/436 | Terrestrial humic | ^30^ |
|  |  |  | P8: <260(355)/434 | Terrestrial humic substances "A" and "C" peaks | ^23^ |
|  |  |  | C1: <260/458 | Humic-like component | ^31^ |
|  |  |  | C3: <250/450 | Humic-like | ^32^ |
|  |  |  | C5: <240/450 | Terrestrial humic-like | ^28^ |
|  |  |  | C1: <240/445 | Terrestrial humic-like | ^33^ |
|  |  |  | C3: <240 (330)/455 | Terrestrial humic-like | ^28^ |

**Table S4**. Photocatalytic degradation of two EEM-PARAFAC components using ZnO under artificial sunlight based on F_max_ values with different pH values, ZnO dosages, and inorganic anions.

| **No.** | **Experimental conditions** | | | **C1** | | | **C2** | | |
| --- | --- | --- | --- | --- | --- | --- | --- | --- | --- |
|  | pH | ZnO dosage (g/L) | Anions  (mM) | Removal (%) | k  (min ^-1^) | R^2^ | Removal (%) | k  (min ^-1^) | R^2^ |
| 1 | 4 | 0.1 | without | 71.75 | 0.0069 | 0.99 | 19.39 | 0.0015 | 0.36 |
| 2 | 4 | 0.2 | without | 99.88 | 0.0244 | 0.94 | 97.93 | 0.0221 | 0.86 |
| 3 | 4 | 0.3 | without | 100.00 | 0.0286 | 0.99 | 98.96 | 0.0290 | 0.94 |
| 4 | 7 | 0.1 | without | 100.00 | 0.0254 | 0.97 | 98.53 | 0.0229 | 0.90 |
| 5 | 7 | 0.2 | without | 100.00 | 0.0348 | 0.94 | 98.97 | 0.0264 | 0.95 |
| 6 | 7 | 0.3 | without | 100.00 | 0.0440 | 0.99 | 99.28 | 0.0309 | 0.93 |
| 7 | 10 | 0.1 | without | 85.22 | 0.0102 | 0.96 | 74.44 | 0.0073 | 0.97 |
| 8 | 10 | 0.2 | without | 100.00 | 0.0266 | 0.98 | 99.19 | 0.0265 | 0.95 |
| 9 | 10 | 0.3 | without | 100.00 | 0.0331 | 0.97 | 99.36 | 0.0282 | 0.98 |
| 10 | 7 | 0.2 | without | 100.00 | 0.0389 | 0.94 | 98.52 | 0.0251 | 0.96 |
| 11 | 7 | 0.2 | [$\mathrm{Cl}^{─}$] = 10mM | 99.92 | 0.0369 | 0.93 | 98.53 | 0.0256 | 0.95 |
| 12 | 7 | 0.2 | [$\mathrm{Cl}^{─}$] = 50mM | 99.90 | 0.0301 | 0.99 | 99.31 | 0.0314 | 0.94 |
| 13 | 7 | 0.2 | [$\mathrm{SO}_{4}^{2─}$] = 10mM | 99.98 | 0.0371 | 0.96 | 98.31 | 0.0249 | 0.96 |
| 14 | 7 | 0.2 | [$\mathrm{SO}_{4}^{2─}$] = 50mM | 99.74 | 0.0345 | 0.99 | 97.47 | 0.0227 | 0.96 |
| 15 | 7 | 0.2 | [$\mathrm{HCO}_{3}^{─}$] = 10mM | 78.78 | 0.0190 | 0.96 | 27.15 | 0.0015 | 1.00 |
| 16 | 7 | 0.2 | [$\mathrm{HCO}_{3}^{─}$] = 50mM | 62.22 | 0.0053 | 0.98 | 6.49 | 0.0006 | 1.00 |

**References**

1. Yan, X., Bao, R. & Yu, S. Effect of inorganic ions on the photocatalytic degradation of humic acid. *Russ. J. Phys. Chem. A* **86**, 1318-1325 (2012).

2. Jayson, G. G., Parsons, B. J. & Swallow, A. J. Some simple, highly reactive, inorganic chlorne derivatives in aqueous solution. *J. Chem. Soc. Faraday Trans. 1 Phys. Chem. Condens. Phases* **69**, 1597-1607 (1973).

3. Grigor’ev, A. E., I. E. Makarov, and A. K. P. Formation of in the bulk of solution during radiolysis of concentrated aqueous solutions of chlorides. *Khimiya Vysok. Ehnergij* **21**, 123-126 (1987).

4. Yu, X. Y. & Barker, J. R. Hydrogen peroxide photolysis in acidic aqueous solutions containing chloride ions. I. Chemical mechanism. *J. Phys. Chem. A* **107**, 1313-1324 (2003).

5. Schwarzenbach, R. P.; Escher, B. I.; Fenner, K.; Hofstetter, T. B.; Johnson, C. A.; Von Gunten, U.; Wehrli, B. The challenge of micropollutants in aquatic systems. *Science* **313**, 1072-1077 (2006).

6. Matthew, B. M.; Anastasio, C. A chemical probe technique for the determination of reactive halogen species in aqueous solution: Part 1 - bromide solutions. *Atmos. Chem. Phys.* **6**, 2423-2437 (2006).

7. McElroy, W. J. A laser photolysis study of the reaction of sulfate(1-) with chloride and the subsequent decay of chlorine(1-) in aqueous solution. *J. Phys. Chem.* **94**, 2435-2441 (1990).

8. Klaning, U. K.; Wolff, T. Laser Flash Photolysis of HClO, ClO^-^ , HBrO, and BrO^-^ in Aqueous Solution. Reaction of Cl^─^ and Br-Atoms. *Berichte der Bunsengesellschaft für physikalische Chemie* **89**, 243-245 (1984).

9. Wu, D., D. Wong, and B. D. B. Evolution of in aqueous NaCl solutions. *J. Photochem.* **14**, 303-310 (1980).

10. Ershov, B. G. Kinetics, mechanism and intermediates of some radiation-induced reactions in aqueous solutions. *Usp. Khim.* **73**, 107-120 (2004).

11. Wang, T. X.; Margerum, D. W. Kinetics of reversible chlorine hydrolysis: Temperature dependence and general-acid/base-assisted mechanisms. *Inorg. Chem.* **33**, 1050-1055 (1994).

12. Santiago, D. E. *et al.* Effect of inorganic ions on the photocatalytic treatment of agro-industrial wastewaters containing imazalil. *Appl. Catal. B Environ.* **156**-**157**, 284-292 (2014).

13. Buxton, G. V, Greenstock, C. L., Helman, W. P. & Ross, A. B. Critical Review of Rate Constants for Reactions of Hydrated Electrons, Hydrogen Atoms and Hydroxyl Radicals (^•^OH/^•^O^−^) in Aqueous Solution. *J. Phys. Chem. Ref. data* **17**, 513-886 (1988).

14. Crittenden, J. C.; Hu, S.; Hand, D. W.; Green, S. A. A kinetic model for H_2_O_2_/UV process in a completely mixed batch reactor. *Water Res.* **33**, 2315-2328 (1999).

15. Dugandžić, A. M. *et al.* Effect of inorganic ions, photosensitisers and scavengers on the photocatalytic degradation of nicosulfuron. *J. Photochem. Photobiol. A Chem.* **336**, 146-155 (2017).

16. Antoniou, M. G., de la Cruz, A. A. & Dionysiou, D. D. Degradation of microcystin-LR using sulfate radicals generated through photolysis, thermolysis and e-transfer mechanisms. *Appl. Catal. B Environ.* **96**, 290-298 (2010).

17. Das, T. N. Reactivity and role of radical in aqueous medium chain oxidation of sulfite to sulfate and atmospheric sulfuric acid generation. *J. Phys. Chem. A* **105**, 9142-9155 (2001).

18. Herrmann, H.; Reese, A.; Zellner, R. Time-resolved UV/VIS diode array absorption spectroscopy of (x=3, 4, 5) radical anions in aqueous solution. *J. Mol. Struct.* **348**, 183-186 (1995).

19. Vu, X. Y., Bao, Z. C. & Barker, J. R. Free Radical Reactions Involving and in the 248 nm Photolysis of Aqueous Solutions Containing and S_2_O_8_^2-^ and Cl^-^. *J. Phys. Chem. A* **108**, 295-308 (2004).

20. Toth, J. E., Rickman, K. A., Venter, A. R., Kiddle, J. J. & Mezyk, S. P. Reaction kinetics and efficiencies for the hydroxyl and sulfate radical based oxidation of artificial sweeteners in water. *J. Phys. Chem. A* **116**, 9819-9824 (2012).

21. Coble, P. G. Characterization of marine and terrestrial DOM in seawater using excitation-emission matrix spectroscopy. *Mar. Chem.* **51**, 325-346 (1996).

22. Murphy, K. R., Ruiz, G. M., Dunsmuir, W. T. M. & Waite, T. D. Optimized parameters for fluorescence-based verification of ballast water exchange by ships. *Environ. Sci. Technol.* **40**, 2357-2362 (2006).

23. Chen, J., LeBoeuf, E. J., Dai, S. & Gu, B. Fluorescence spectroscopic studies of natural organic matter fractions. *Chemosphere* **50**, 639-647 (2003).

24. Baghoth, S. A., Sharma, S. K. & Amy, G. L. Tracking natural organic matter (NOM) in a drinking water treatment plant using fluorescence excitation-emission matrices and PARAFAC. *Water Res.* **45**, 797-809 (2011).

25. He, W. & Hur, J. Conservative behavior of fluorescence EEM-PARAFAC components in resin fractionation processes and its applicability for characterizing dissolved organic matter. *Water Res.* **83**, 217-226 (2015).

26. Ishii, S. K. L. & Boyer, T. H. Behavior of reoccurring parafac components in fluorescent dissolved organic matter in natural and engineered systems: A critical review. *Environ. Sci. Technol.* **46**, 2006-2017 (2012).

27. Phong, D. D. & Hur, J. Insight into photocatalytic degradation of dissolved organic matter in UVA/TiO_2_ systems revealed by fluorescence EEM-PARAFAC. *Water Res.* **87**, 119-126 (2015).

28. Ahn, Y. *et al.* Characteristics and fate of natural organic matter during UV oxidation processes. *Chemosphere* **184**, 960-968 (2017).

29. Timko, S. A. *et al.* Depth-dependent photodegradation of marine dissolved organic matter. *Front. Mar. Sci.* **2**, 1-13 (2015).

30. Stedmon, C. A., Markager, S. & Bro, R. Tracing dissolved organic matter in aquatic environments using a new approach to fluorescence spectroscopy. *Mar. Chem.* **82**, 239-254 (2003).

31. Yamashita, Y., Jaffé, R., Maie, N. & Tanoue, E. Assessing the dynamics of dissolved organic matter (DOM) in coastal environments by excitation emission matrix fluorescence and parallel factor analysis (EEM-PARAFAC). *Limnol. Oceanogr.* **53**, 1900-1908 (2008).

32. Yao, X. *et al.* Resolving the variability of CDOM fluorescence to differentiate the sources and fate of DOM in Lake Taihu and its tributaries. *Chemosphere* **82**, 145-155 (2011).

33. Wu, F. C., Evans, R. D. & Dillon, P. J. Separation and characterization of NOM by high-performance liquid chromatography and on-line three-dimensional excitation emission matrix fluorescence detection. *Environ. Sci. Technol.* **37**, 3687-3693 (2003).
